# Supplementary material for: Ten-eleven translocation 1 mediated-DNA hydroxymethylation is required for myelination and remyelination in the mouse brain
Source: Nat Commun. 2021 Aug 24;12:5091. doi: 10.1038/s41467-021-25353-5 (PMC8385008; doi:10.1038/s41467-021-25353-5)
Supplement: Supplementary file 1 — Supplementary information [file 41467_2021_25353_MOESM1_ESM.pdf]

Supplementary Information

Ten-eleven translocation 1 Mediated-DNA Hydroxymethylation is Required for Myelination and Remyelination in the Mouse Brain

Ming Zhang[1], Jian Wang[1], Kaixiang Zhang[1], Guozhen Lu[1], Yuming Liu[1], Keke Ren[1], Wenting Wang[1], Dazhuan Xin[2], Lingli Xu[3], Honghui Mao[1], Junlin Xing[1], Xingchun Gao[4], Weilin Jin[5], Kalen Berry[2], Katsuhiko Mikoshiba[6], Shengxi Wu[1,*], Q. Richard Lu[2,*] and Xianghui Zhao[1,*]

Supplementary figures:

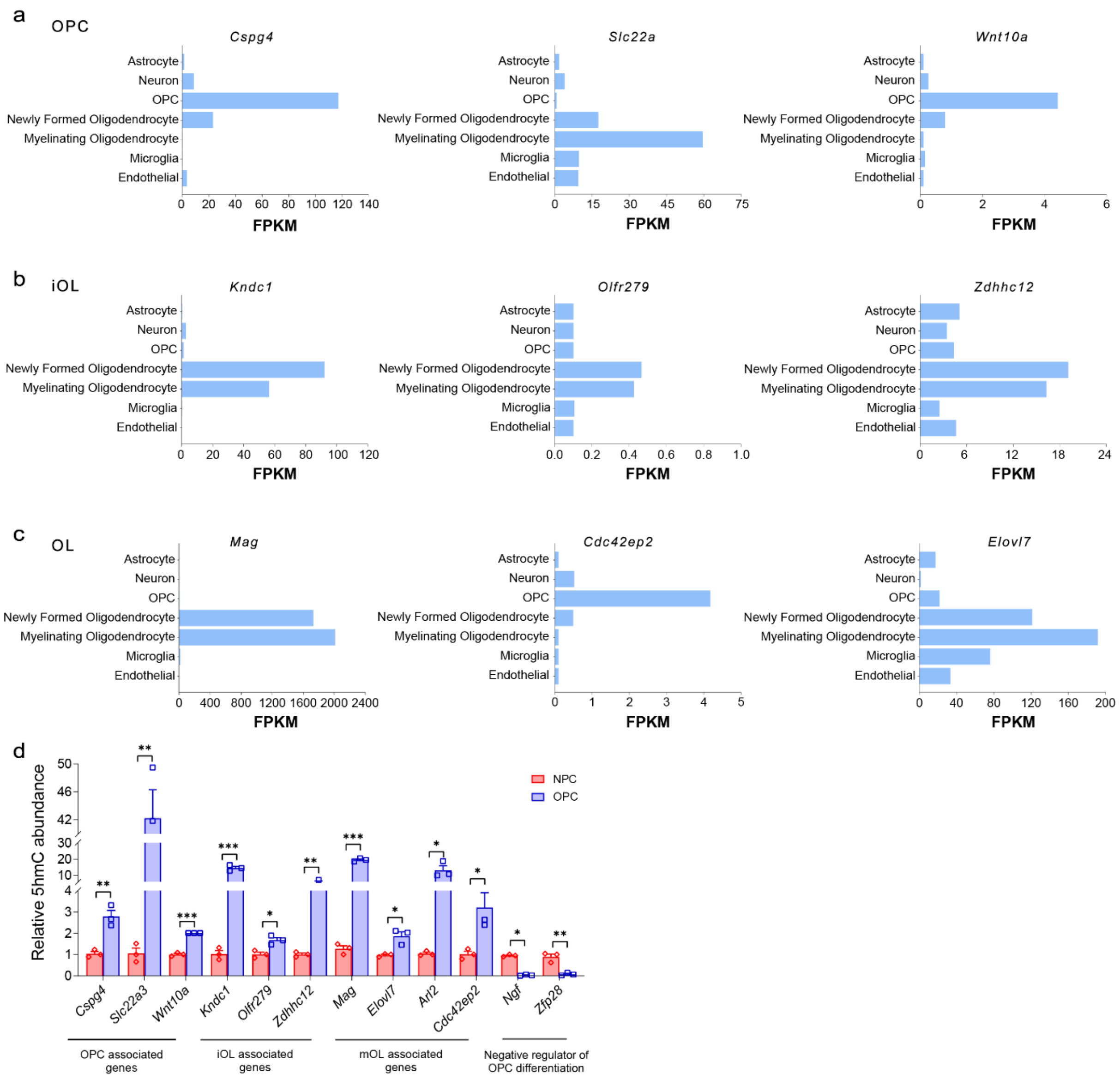

Supplementary Fig. 1 Distribution pattern of genomic 5hmCs is associated with the transition from NPCs to OPCs

(a-c) Examples of OL lineage-specific genes expressed in different OL stages. Fragments per kilobase of transcript per million mapped reads (FPKMs) of representative genes highly expressed in OPCs (a), iOLs (b), and OLs (c). Diagrams were made from http://jiaqianwulab.org/braincell/RNASeq.html.

(d) The level of 5hmC in certain gene loci is identified by qPCR assay. Validation of loci specific 5hmC modification in OPC and NPC cultures by qPCR analysis of OPC associated genes, immature OLs (iOL) associated genes, mOL associated genes and negative regulators of OPC differentiation. Data are Means ± SEM (n = 3 independent cultures per group). *, p<0.05, **, p<0.01, ***, p<0.001, compared to NPC. Two-tailed unpaired t test. *Cspg4*: t = -5.867, df = 4, p = 0.004; *Slc22a3*: t = -10.100, df = 4, p = 0.001; *Wnt10a*: t = -20.501, df = 4, p = 0.000033; *Kndc1*: t = -13.045, df = 4, p = 0.000199; *Olfr279*: t = -4.221, df = 4, p = 0.013; *Zdhhc12*: t = -5.173, df = 4, p = 0.007; *Mag*: t = -32.333, df = 4, p = 0.000005; *Elovl7*: t = -4.274, df = 4, p = 0.013; *Arl2*: t = -4.319, df = 4, p = 0.012; *Cdc42ep2*: t = -3.081, df = 4, p = 0.037; *Ngf*: t = 27.294, df = 4, p = 0.037; *Zfp28*: t = 5.877, df = 4, p = 0.004.

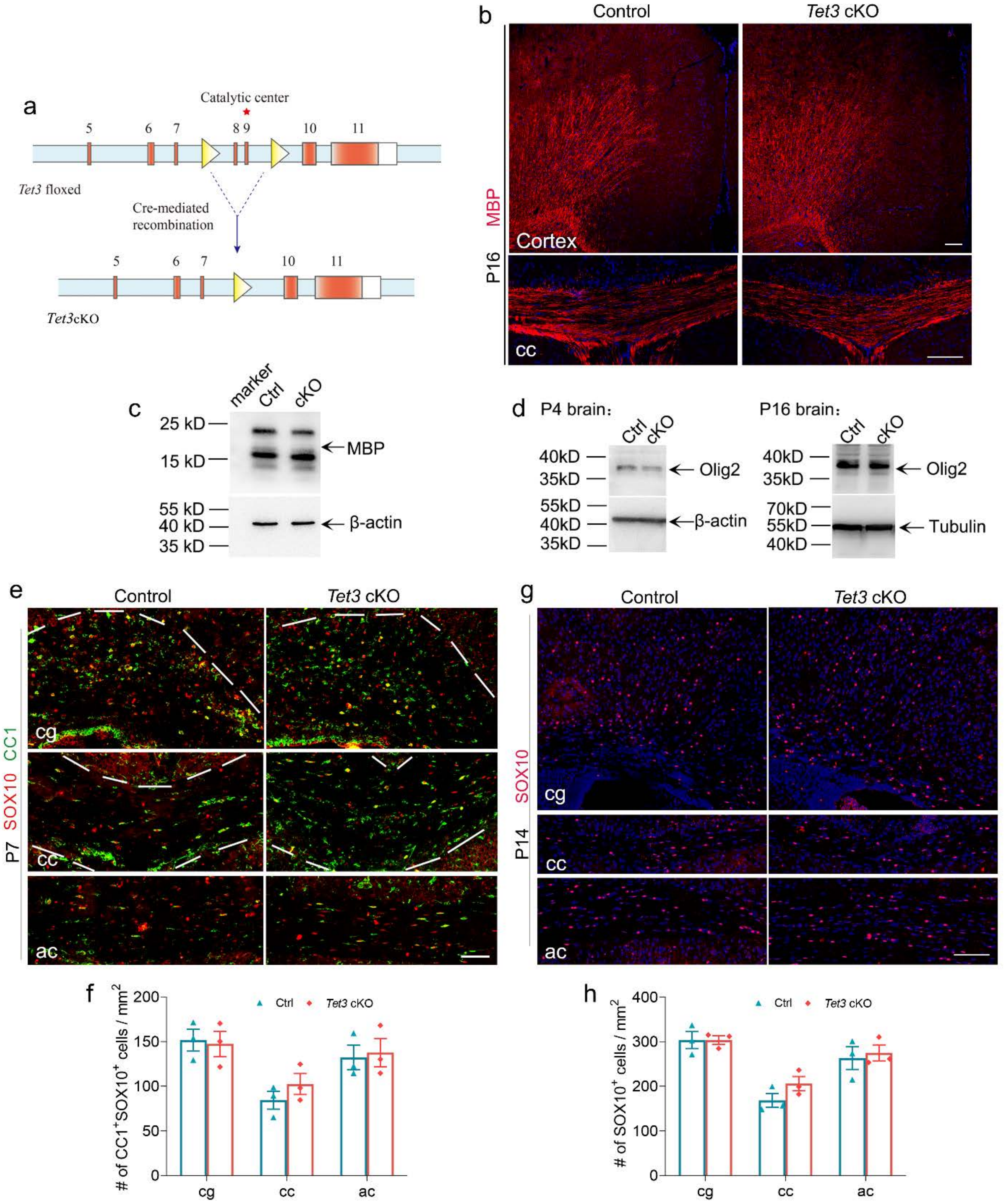

Supplementary Fig. 2 Deletion of *Tet3* does not cause significant effects on myelination

(a) Schematic diagram of Cre-mediated excision of floxed *Tet3* exons encoding the catalytic center.

(b) Representative image of MBP immunostaining of postnatal day 16 (P16) brains from control and *Tet3* cKO mice. Scale bar, 100 μm.

(c) Western blot assay for MBP expression in P16 control (Ctrl) and *Tet3* cKO (cKO) brains. β-actin was detected as loading control.

(d) Western blot assay for OLIG2 expression in P4 and P16 control and *Tet3* cKO brains. β-actin and Tubulin was detected as loading control, respectively.

(e) Representative images of three regions of P7 control and *Tet3* cKO brains stained for SOX10 and CC1. Scale bar, 100 μm. cc; corpus callosum; cg, cingulum; ac, anterior commissure.

(f) Density of $SOX10^{+}CC1^{+}$ cells in P7 control and *Tet3* cKO brains. Data are Means ± SEM (*n* =

3 animals per group). Two-tailed unpaired *t* test, cg: $t = 0.226$, $df = 4$, $p = 0.833$; cc: $t = -0.170$, $df = 4$, $p = 0.307$; ac: $t = -0.257$, $df = 4$, $p = 0.810$.

(g) Representative images of three regions of P14 control and *Tet3* cKO brains stained for SOX10. Scale bar, 100 μm.

(h) Quantification of $SOX10^+$ cells in P14 control and *Tet3* cKO brains. Data are Means ± SEM ($n = 3$ animals per group). Two-tailed unpaired *t* test, cg: $t = 0.012$, $df = 4$, $p = 0.991$; cc: $t = -1.684$, $df = 4$, $p = 0.168$; ac: $t = -0.380$, $df = 4$, $p = 0.723$.

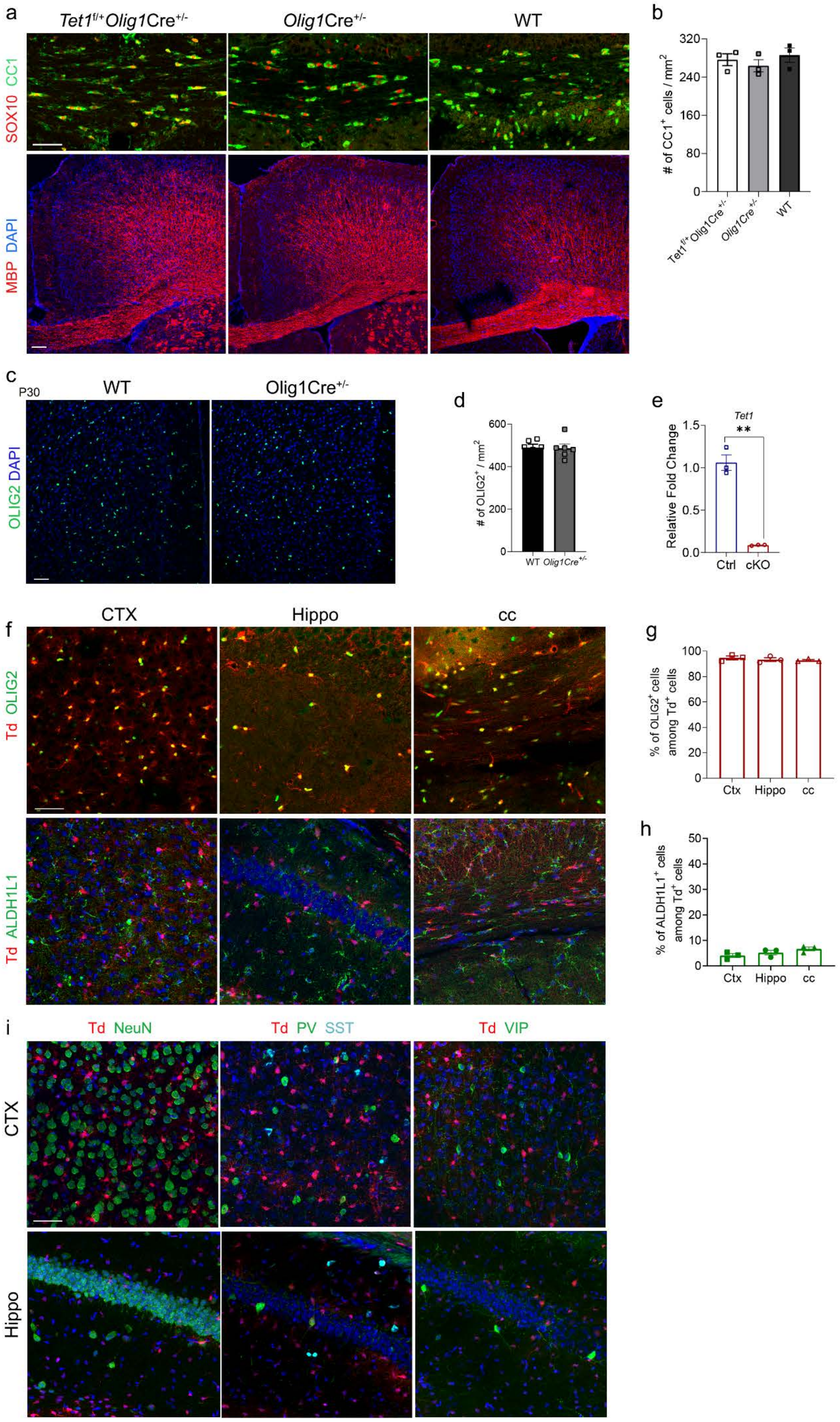
a
Tet1f/+Olig1Cre+/-
Olig1Cre+/-
WT
SOX10 CC1
MBP DAPI
b
# of CC1+ cells / mm2
320
240
160
80
0
Tet1f/+Olig1Cre+/-
Olig1Cre+/-
WT
c
P30
WT
Olig1Cre+/-
OLIG2 DAPI
d
# of OLIG2+ / mm2
600
400
200
0
WT
Olig1Cre+/-
e
Tet1
**
Relative Fold Change
1.5
1.0
0.5
0.0
Ctrl
cKO
f
CTX
Hippo
cc
Td OLIG2
Td ALDH1L1
g
% of OLIG2+ cells among Td+ cells
100
80
60
40
20
0
Ctx
Hippo
cc
h
% of ALDH1L1+ cells among Td+ cells
50
40
30
20
10
0
Ctx
Hippo
cc
i
Td NeuN
Td PV SST
Td VIP
CTX
Hippo

Supplementary Fig. 3 OL differentiation and myelination are similar among *Tet1* heterozygous mice, Cre control, and wild-type mice, and *Olig1*-Cre is predominantly restricted to OL lineage

(a) Representative immunostaining of P14 brains from indicated mice for mature OL marker CC1, OL lineage marker SOX10, and myelin protein MBP. Scale bar, 50 μm.

(b) Quantification of $CC1^+$ cells in P14 brains of indicated mice. Data are Means ± SEM (*n* = 3 animals per group). One way ANOVA, $F_{(2,6)} = 0.688$, $p = 0.538$.

(c) Representative immunostaining of P30 cortex from indicated mice for OLIG2. Scale bar, 50 μm.

(d) Quantification the density of $OLIG2^+$ cells reveal no significant difference between indicated mice. Data are Means ± SEM (*n* = 6 animals per group). Two-tailed unpaired *t* test, $t = 0.076$, $df = 10$, $p = 0.941$.

(e) Real-time PCR quantification of *Tet1* mRNA in OPCs purified from *Tet1* cKO brain. Data are Means ± SEM (*n* = 3 independent cultures each performed in triplicate). Two-tailed unpaired separate variance estimation *t* test, **, $t = 10.61$, $df = 2.005$, $p = 0.0087$.

(f) Representative immunostaining of OLIG2 and ALDH1L1 in cortex (CTX), Hippocampus (Hippo) and corpus callosum (cc) in P21 *Olig1*Cre-tdTomato mice. Scale bar, 50 μm.

(g) Quantification the percentage of $OLIG2^+$ cells among Td^+ cells from different brain region. Data are Mean ± SEM (*n* = 3 animals each group). One way ANOVA, $F_{(2,6)} = 4.465$, $p = 0.442$.

(h) Quantification the percentage of $ALDH1L1^+$ cells among Td^+ cells from different brain region. Data are Mean ± SEM (n=3 animals each group). Kruskal-Wallis H test, $\chi^2=3.200$, $df = 2$, $p = 0.202$.

(i) Representative immunostaining of NeuN, PV, SST and VIP in P21 cortex and hippocampus of *Olig1*Cre-tdTomato mice. Rare co-labeling of NeuN, PV, SST and VIP with Td was observed. Scale bar, 50 μm.

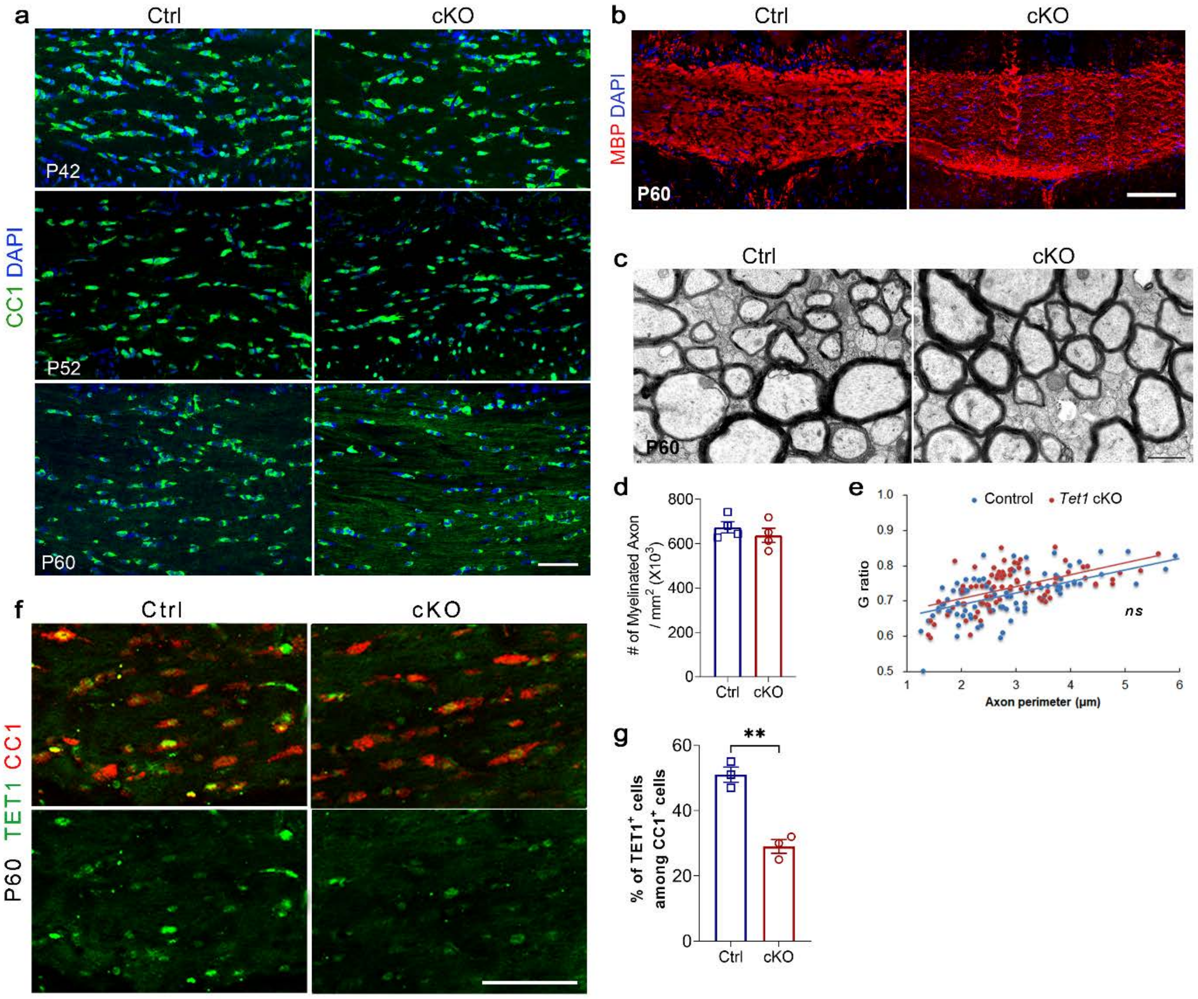

Supplementary Fig. 4 Normal myelination in adult *Tet1* cKO mice

(a) Representative immunostaining images of corpus collosum from P42, P52 and P60 controls and *Tet1* cKO mice stained for CC1. Scale bar, 50 μm.

(b) Representative immunostaining images of corpus collosum from P60 controls and *Tet1* cKO mice stained for MBP. Scale bar, 50 μm.

(c) Representative electron micrographs of corpus collosum from control and *Tet1* cKO mice at P60. Scar bar, 0.5 μm.

(d) Quantification the numbers of myelinated axons in defined areas from P60 control and *Tet1* cKO mice. Data are Means ± SEM (n = 3 animals per group). Two-tailed unpaired t test, t = 0.0891, df = 6, p = 0.407.

(e) G ratios versus axonal perimeters for control and *Tet1* cKO mice at P60. *ns*; not significant, Friedman M test, χ^2 = 2.390, df = 1, $p_{\text{between group}}$ = 0.122 (> 80 myelinating axon counts from 3 animals each genotype).

(f) Representative images for TET1 and CC1 expression in corpus callosum of P60 mice. Scale bar, 50 μm.

(g) Quantification of TET1$^+$ cells among CC1$^+$ cells in control and *Tet1* cKO mice. Data are presented as Mean ± SEM (n = 3 animals per group). **, Two-tailed unpaired t test, t = 7.706, df = 6, p = 0.002.

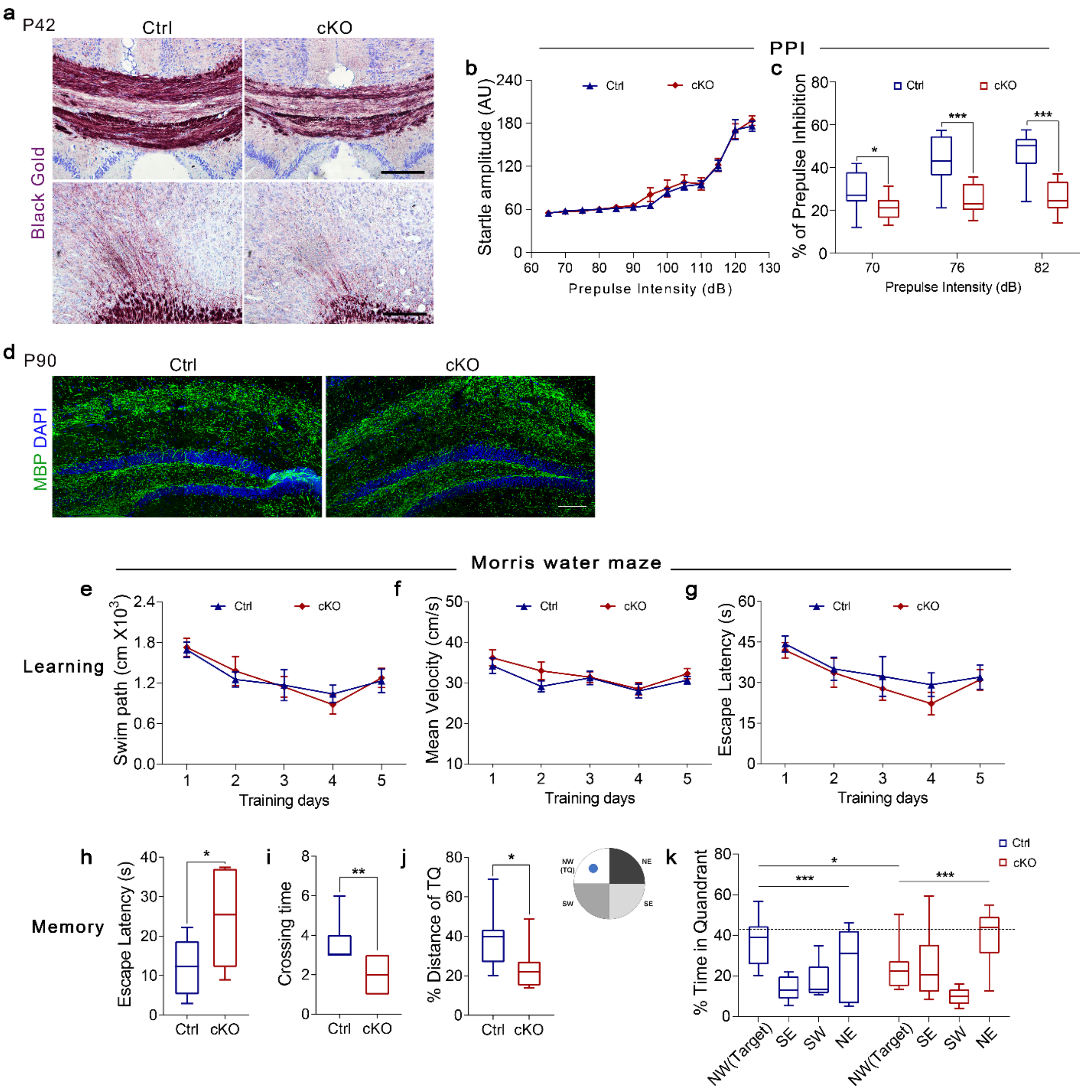

Supplementary Fig. 5 Behavioral deficiency in *Tet1* cKO mice

(a) Black gold staining shows myelin deficiency in P42 *Tet1* cKO mice. Scar bar, 100 μm.

(b) Similar baseline startle response was observed at multiple pulse intensities ranging from 65 to 125 dB in *Tet1* cKO mice and control littermates. Data were expressed as Mean ± SEM (n = 13 for control mice and n = 14 for *Tet1* cKO mice). RMANOVA, $F_{\text{between group (1,25)}} = 0.1836$, p = 0.6720, $F_{\text{within group (12,300)}} = 144.6$, p <0.0001. AU, arbitrary units.

(c) Impaired PPI in *Tet1* cKO mice for 70, 76 and 82 dB prepulse sound level. Boxes were expressed as quartiles (median, 25th and 75th percentile), whiskers were defined as minima and maxima (n = 13 for control mice and n =14 for *Tet1* cKO mice). Two-tailed unpaired t test, 70dB: t = 2.724, df = 25, p = 0.012; 76dB: t = 4.913, df = 25, p = 0.000047; 82dB: t = 5.611, df = 25, p = 0.000008.

(d) Representative MBP immunostaining for P90 adult mice in the hippocampus reveals comparable myelin between groups. Scar bar, 100 μm.

(e-g) Working memory impairment in *Tet1* cKO adult mice was revealed in the Morris water maze. *Tet1* mutant show normal spatial reference memory acquisition during 5-day training, indicated by the swimming distance (e), the mean swimming velocity (f) and the escape latency to the hidden platform (g). Data are expressed as Mean ± SEM (n = 8 animals per group, four trials per day). RMANOVA test: (e) $F_{\text{between group (1,14)}} = 0.001$, p = 0.976, $F_{\text{within group (4,56)}} = 7.260$, p = 0.000088;

(f) $F_{\text{between group }(1,14)}$ = 1.100, p = 0.312, $F_{\text{within group }(4,56)}$ = 8.034, p = 0.000034; (g) $F_{\text{between group }(1,14)}$ = 0.765, p = 0.397, $F_{\text{within group }(4,56)}$ = 6.571, p = 0.000208.
(h-k) Probe trial for short-term memory retention was carried out 24hr after the last training. Compared to the control group, *Tet1* cKO mice spent longer time in searching the removed platform position (h) and crossed less times through the position (i). The reduced swimming distance (j) and swimming time in the target quadrants (TQ) indicate impaired spatial reference memory retention in *Tet1* mutant (k). NW, northwest; SE, southeast; SW, southwest; NE, northeast. Boxes were expressed as quartiles (median, 25th and 75th percentile), whiskers were defined as minima and maxima (n = 8 animals per group). (h): *, Two-tailed unpaired t test, t = -2.650, df = 14, p = 0.019; (i): **, Mann-Whitney U test, z = -2.711, p = 0.007; (j): *, Mann-Whitney U test, z =-2.205, p = 0.027; (k): Two-tailed unpaired t test for MW quadrant between Ctrl and cKO group, *, t = 2.436, df = 14, p = 0.029. One way ANOVA for % time in different quadrant. Ctrl: ***, $F_{(3,28)}$ = 8.153, p = 0.000467. NW vs. SE, ***, q = 24.4375, p = 0.000461; NW vs. SW, **, q = 19.58000, p = 0.005; NW vs. NE, q = 10.8500, p = 0.198; SE vs. SW, q = 4.85750, p = 0.798; SE vs. NE, q = -13.58150, p = 0.073; SW vs. NE, q = -8.73000, p = 0.372. cKO: ***, $F_{(3,28)}$ = 7.685, p = 0.00067. NW vs. SE, q = -0.47375, p = 1.0; NW vs. SW, q = 14.54850, p = 0.116; NW vs. NE, q = -15.47500, p = 0.086; SE vs. SW, q = 15.01625, p = 1.0; SE vs. NE, q = -15.00125, p = 1.0; SW vs. NE, ***, q = -30.01750, p = 0.000266.

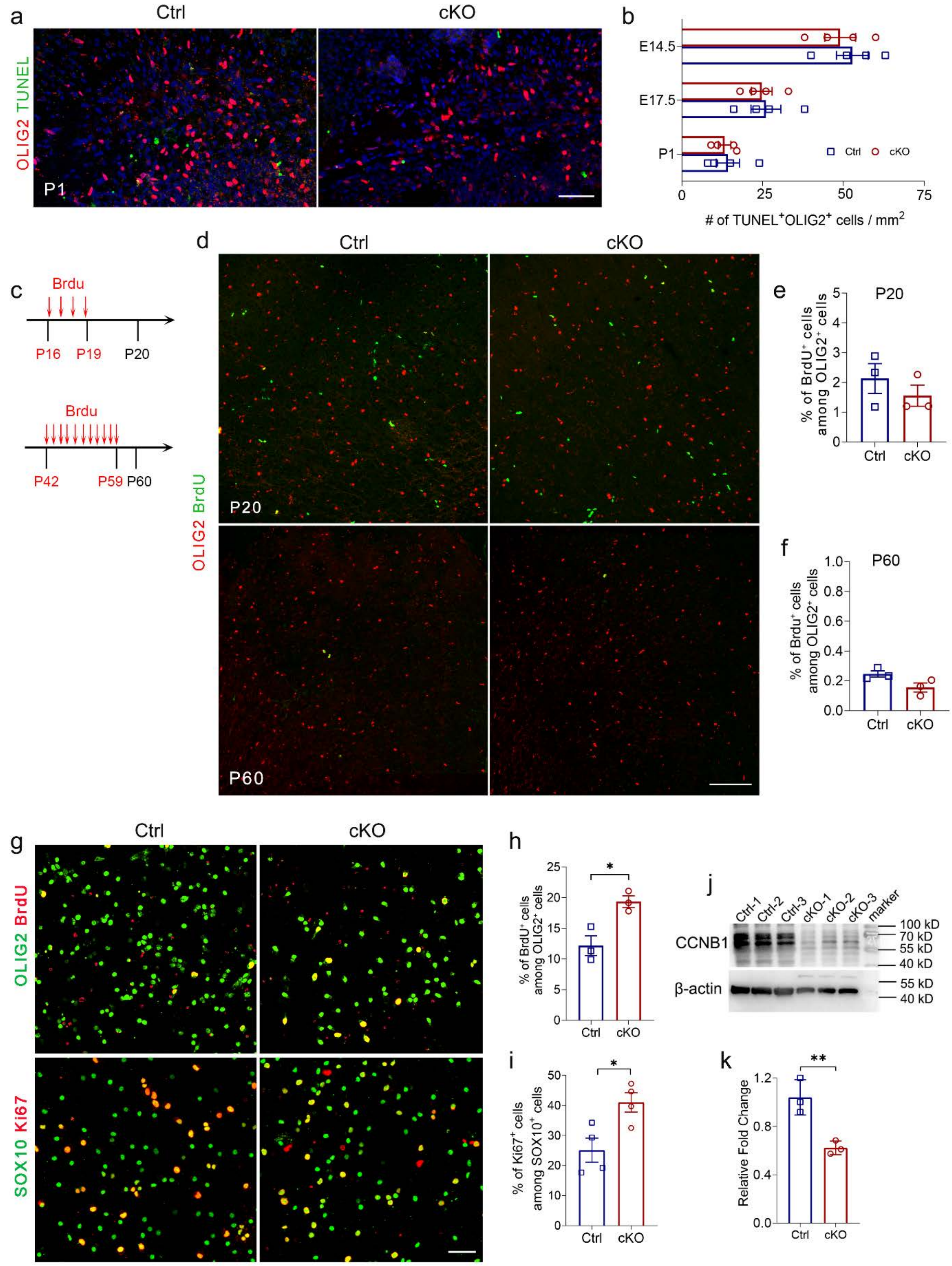

Supplementary Fig. 6 Normal OPC apoptosis and increased proliferation in OPCs purified from *Tet1* cKO mice

(a) Representative images of P1 control and *Tet1* cKO brains stained for OLIG2 after TUNEL staining. Scale bar, 50 μm.

(b) Quantification of $TUNEL^+$ $OLIG2^+$ cells in control and *Tet1* cKO brains from E14.5 to P1 stages. Data are Means ± SEM (n = 4 animals each group). No significant differences were observed.

Two-tailed unpaired *t* test, E14.5: *t* = 0.246, *df* = 6, *p* = 0.714; E17.5: *t* = 0.223, *df* = 6, *p* = 0.831; P1: *t* = 0.542, *df* = 6, *p* = 0.604.
(c) Diagram showing BrdU injection to label proliferating cells in P20 and P60 mice.
(d) Representative images of BrdU and OLIG2 immunostaining in P20 and P60 cortex. Scale bar, 100 μm.
(e-f) Quantification the percentage of $BrdU^+$ cells among $OLIG2^+$ cells, suggesting no difference between control and *Tet1* cKO mice at indicated stages. Data are Means ± SEM (*n* = 3 animals each group). (e): Mann-Whitney U test, *z* = -0.655, *p* = 0.513; (f): Two-tailed unpaired *t* test, *t* = 2.444, *df* = 4, *p* = 0.071.
(g) Representative images of double immunostaining OPC cultures with BrdU / OLIG2, or Ki67 / SOX10 to illustrate the proliferating cells in OL lineage. Scale bar, 50 μm.
(h-i) Comparison the proportion of $BrdU^+$ (h) and $Ki67^+$ cells (i) among OPCs between control and *Tet1* cKO mice. Note the increased number of proliferating OPCs in *Tet1* cKO group. Data are Mean ± SEM (*n* = 3 independent experiments each group). (h): *, Two-tailed unpaired *t* test, *t* = -3.832, *df* = 4, *p* = 0.019; (i): *, Two-tailed unpaired *t* test, *t* = -3.093, *df* = 6, *p* = 0.021.
(j) Western blot assay for the expression of CCNB1 in purified control and *Tet1* cKO OPC cultures. β-actin is used for loading control.
(k) Histogram shows fold changes measured by densitometry in *Tet1* cKO group relative to control after normalization to β-actin levels. Data are Mean ± SEM (*n* = 3 independent cultures). **, Two-tailed unpaired *t* test, *t* = 4.638, *df* = 4, *p* = 0.0098.

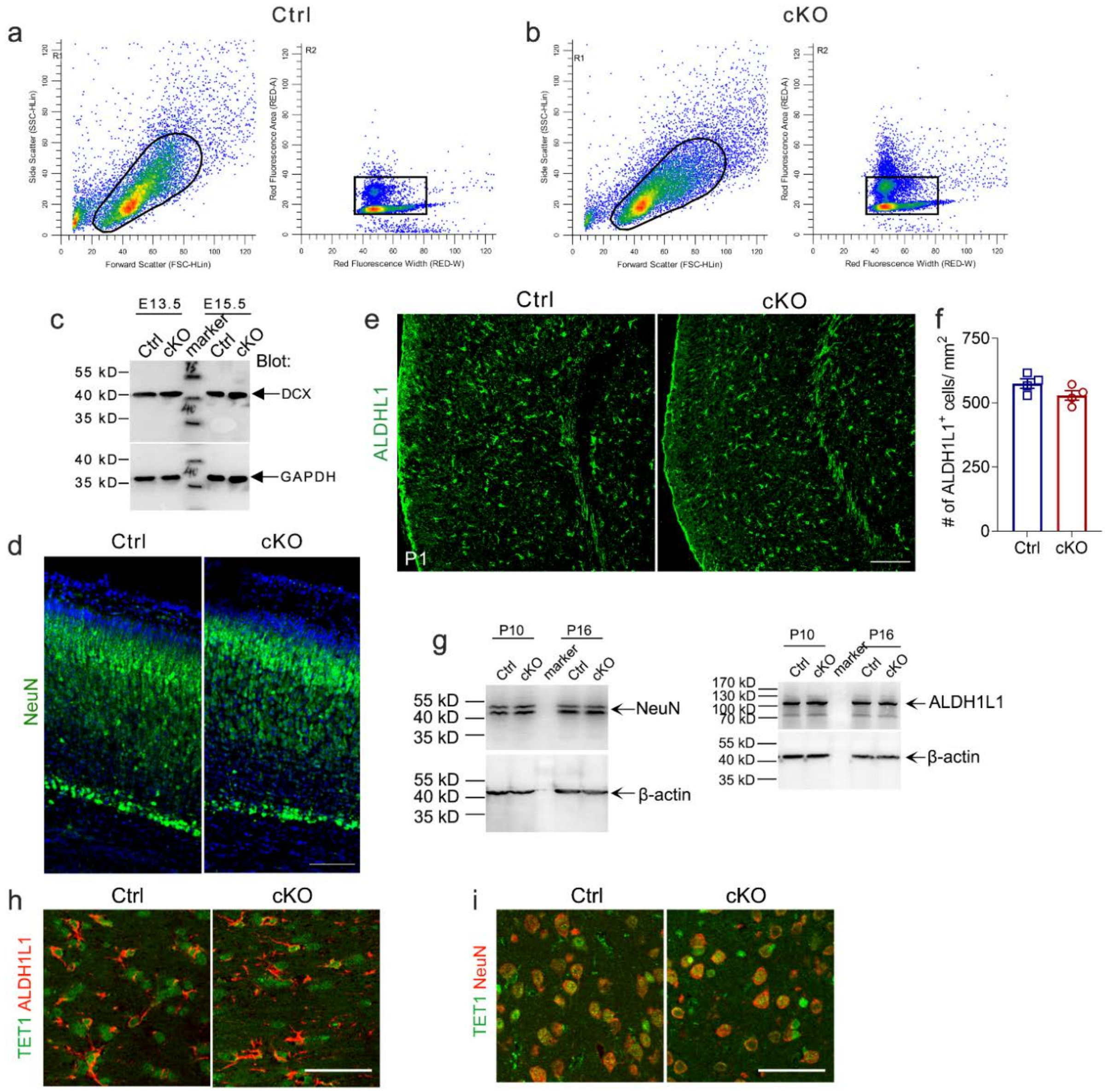

Supplementary Fig. 7 Normal astrocytes and neurons development in *Tet1* cKO mice

(a-b) Representative gating strategies for flow-cytometry analysis of cell cycle in purified OPCs from Control (a) and *Tet1* cKO (b) mice. The sorting strategy is as follows: on the first sort, the major cell groups is included by drawing a gate around cells with 1.0-7.5 K forward (FSC-HLin) and side (SSC-HLin) scatter. On the second sort, the single cell was gated in 3.0K-5.0K Red Fluorescence Width and 1.5K-6.0K Area. By looking at forward scatter vs width, we exclude cellular debris and large cell masses.

(c) Western blot assay for expression of DCX in control and *Tet1* cKO E13.5 and E15.5 brains. GAPDH was detected as a loading control.

(d) Representative images of P1 control and *Tet1* cKO brains immunostained for NeuN. Scale bar, 100 μm.

(e) Representative images of P1 control and *Tet1* cKO S1 cortex stained for ALDH1L1. Scale bar, 100 μm.

(f) Quantification of ALDH1L1^{+} cells from P1 control and *Tet1* cKO S1 cortex. Data are Means ± SEM (n = 3 animals per group). Two-tailed unpaired t test, t = 1.755, df = 6, p = 0.130.

(g) Western blot assay for the expression of ALDH1L1 and NeuN in the brain samples from postnatal control and *Tet1* cKO mice; β-actin was detected as a loading control.

(h-i) Representative images of P10 control and *Tet1* cKO brains immunostained for TET1/ALDH1L1 (h) and TET1/NeuN (i). Scale bar, 50 μm.

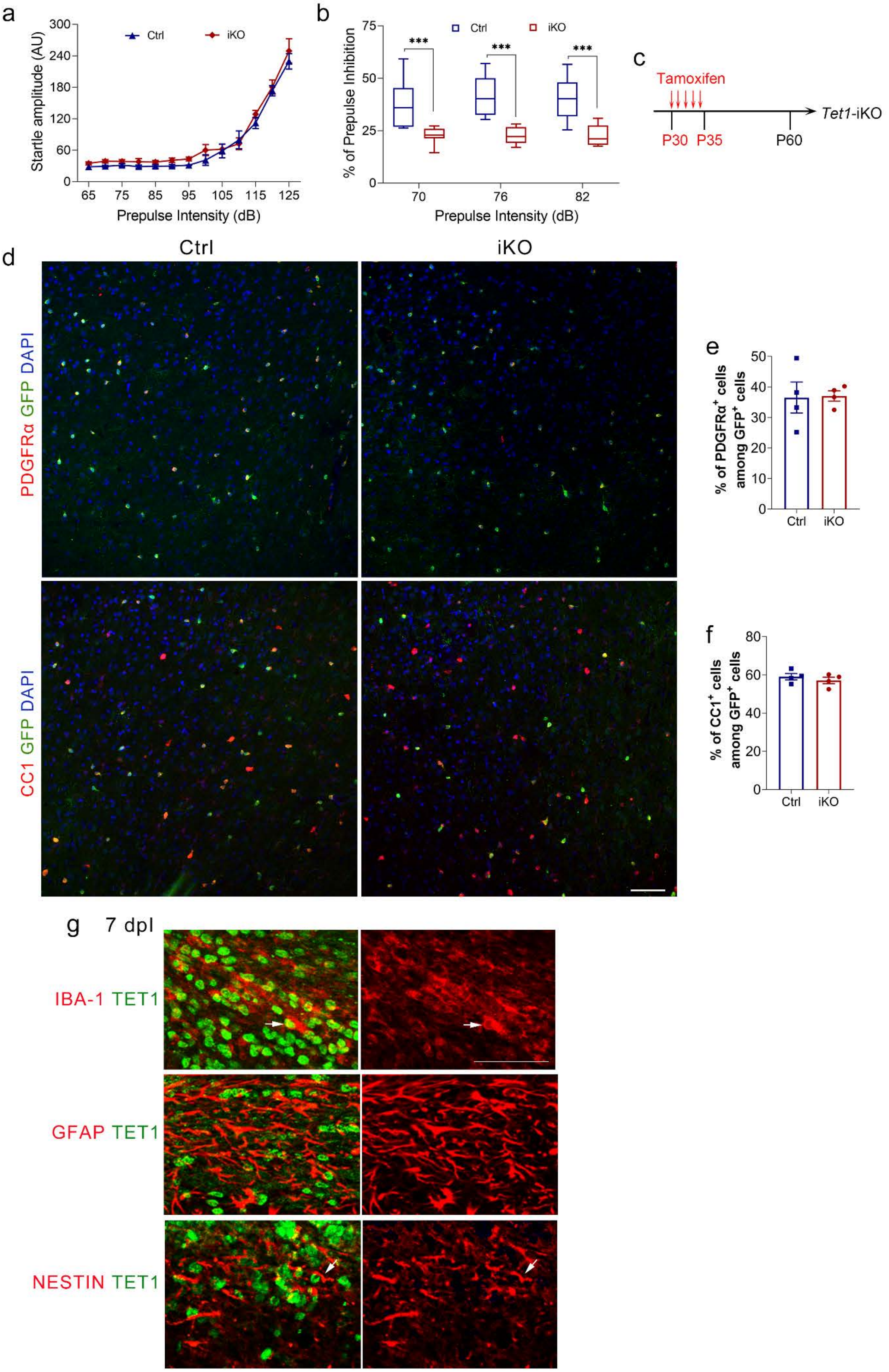
a
Startle amplitude (AU)
Ctrl
iKO
Prepulse Intensity (dB)
b
% of Prepulse Inhibition
***
c
Tamoxifen
P30
P35
P60
Tet1-iKO
d
Ctrl
iKO
PDGFRα GFP DAPI
CC1 GFP DAPI
e
% of PDGFRα+ cells among GFP+ cells
f
% of CC1+ cells among GFP+ cells
g
7 dpl
IBA-1 TET1
GFAP TET1
NESTIN TET1

Supplementary Fig. 8 Impaired PPI in *Tet1* iKO (*NG2-CreER; Tet1$^{flox/flox}$*) mice and OPC fate in adult *Tet1* iKO-GFP mice

(a) Similar baseline startle response was observed at multiple pulse intensities ranging from 65 to 125 dB in *Tet1* iKO mice and control littermates. Data were expressed as Mean ± SEM (n = 8 for *Tet1* cKO and control mice). RMANOVA, $F_{\text{between group (1,14)}} = 3.589$, $p = 0.079$, $F_{\text{within group (2.747,41.21)}} = 458.2$, $p < 0.0001$. AU, arbitrary units.

(b) Impaired PPI in *Tet1* iKO mice for 70, 76 and 82 dB prepulse sound level. Boxes were expressed as quartiles (median, 25th and 75th percentile), whiskers were defined as minima and maxima (n = 11 for *Tet1* iKO and control mice). ***, Two-tailed unpaired separate variance estimation t test, 70dB: $t = 4.584$, $df = 12.804$, $p = 0.00053$; 76dB: $t = 6.447$, $df = 13.771$, $p = 0.000017$; 82dB: $t = 5.386$, $df = 14.432$, $p = 0.000086$.

(c) Diagram showing Tamoxifen administration to induce Cre recombination from P30 in *Tet1* iKO mice with GFP Cre reporter. Mice were sacrificed for analysis at P60.

(d) Representative double immunostaining for PDGFRα / GFP and CC1 / GFP in the cortex of P60 *Tet1* iKO, R26-YFP mice. Scale bar, 100 μm.

(e) Quantification the percentage of PDGFRα$^+$ cells in GFP$^+$ cells in control and *Tet1* iKO mice. Data are Mean ± SEM (n = 4 of animals each group). Two-tailed unpaired t test, $t = -0.102$, $df = 6$, $p = 0.922$.

(f) Quantification the percentage of CC1$^+$ cells in GFP$^+$ cells in control and *Tet1* iKO mice. Data are Mean ± SEM (n = 4 of animals each group). Two-tailed unpaired t test, $t = 0.827$, $df = 6$, $p = 0.440$.

(g) TET1 expression can be detected in microglia and NPCs, not in astrocytes, in LPC lesion site. Representative double immunostaining of TET1 with microglia marker IBA1, astrocyte marker GFAP and neural progenitor maker NESTIN in the lesion site. TET1 expression in IBA-1$^+$ cells and few NESTIN$^+$ cells are indicated by the arrows. Scale bar, 100 μm.

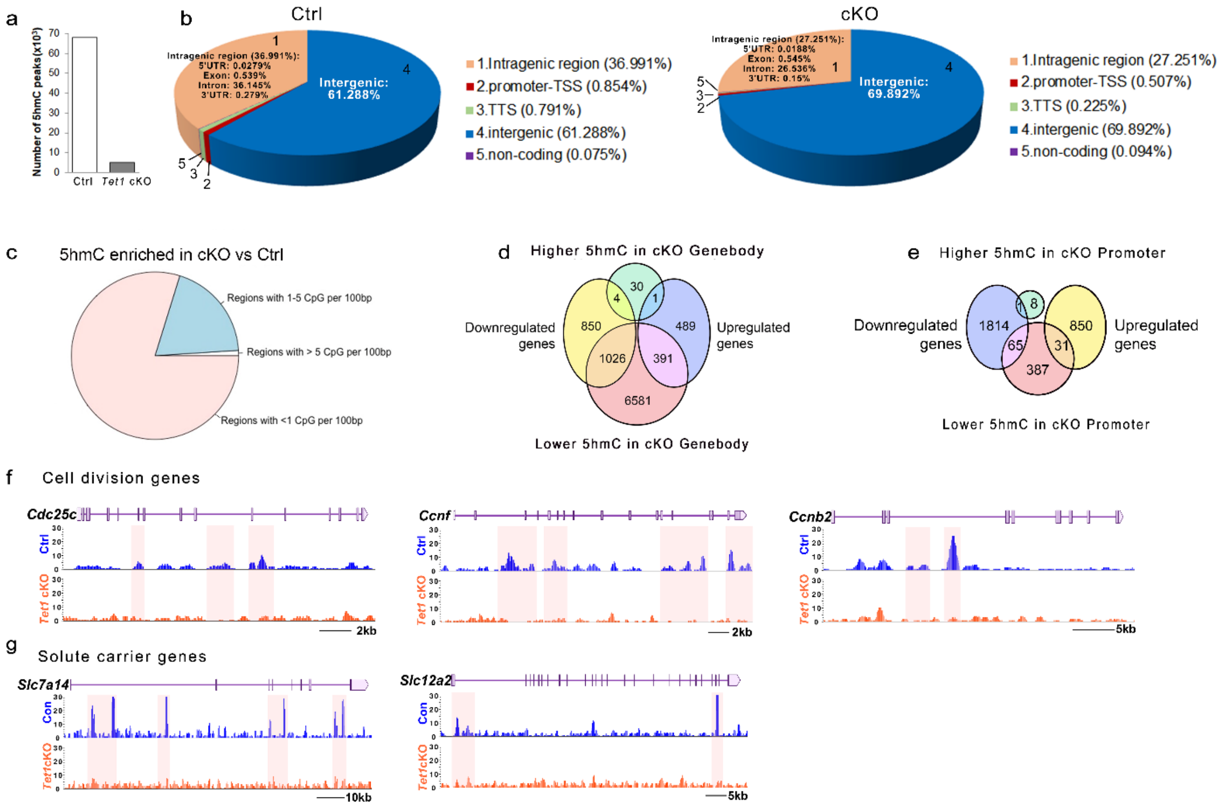

Supplementary Fig. 9 5hmC distribution in the genome of OPCs is altered in *Tet1* cKO mice

(a) The number of total 5hmC peaks from hMeDIP-seq in control and *Tet1* cKO mice.
(b) Genomic distribution of 5hmC in OPCs from control and *Tet1* cKO mice.
(c) Proportion of differentially hydroxymethylated regions with different CpG densities.
(d) Venn diagram of the overlap between differentially hydroxymethylated peaks at gene bodies and differentially expressed genes.
(e) Venn diagram of the overlap between genes differentially hydroxymethylated at promoters and differentially expressed genes.
(f) Representative 5hmC peaks of cell division genes in OPCs derived from control (blue) and *Tet1* cKO (orange) mice.
(g) Representative 5hmC peaks of *Slc* family genes in OPCs derived from control (blue) and *Tet1* cKO (orange) mice.

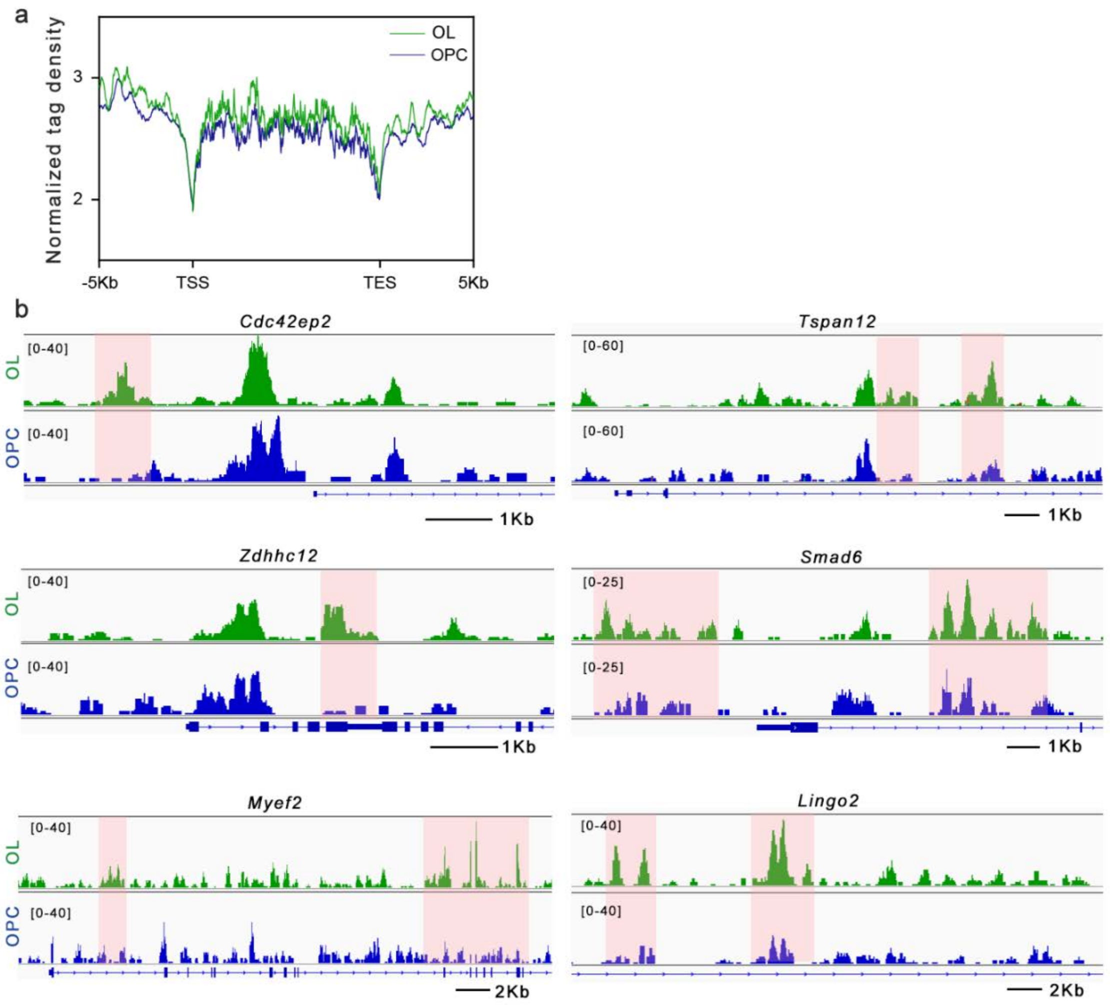

Supplementary Fig. 10 Comparation 5hmC land scape between OPC and OL.
(a) Normalized 5hmC tag density distribution from normal OPCs and OLs.
(b) Snapshots of 5hmC profiles of representative genes with hyper-hydroxyemethylated regions in OLs (green) compared with OPCs (blue). Pink boxes label the hyper-5hmC region in OLs.

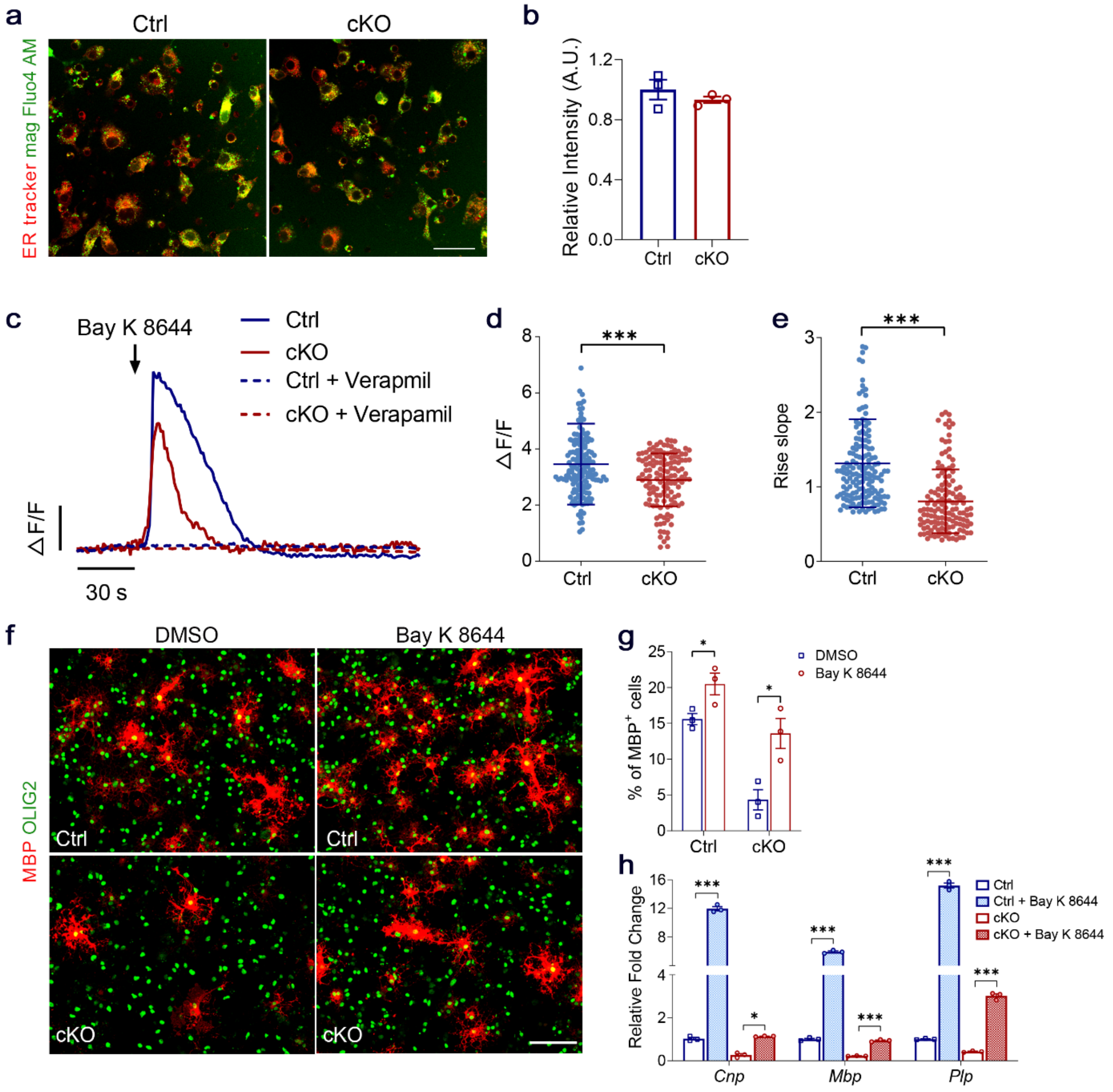

Supplementary Fig. 11 Activating calcium rescues the impaired differentiation in *Tet1* depleted OPCs

(a) Representative images of living OPCs from control and *Tet1* cKO mice loaded with mag Fluo4 AM (Green) and ER tracker (Red). Scale bar, 40 μm.

(b) Relative fluorescence intensity of mag Fluo4 AM measured by spectrofluorometer from control and *Tet1* cKO OPC cultures. Data are Means ± SEM (n = 3 independent cultures), Two-tailed unpaired t test, t = 1.933, df = 4, p = 0.125.

(c) Representative traces of Fluo4 intensity of OPCs from control and *Tet1* cKO mice following application of Bay K 8644 and Verapamil.

(d) Amplitude changes ($\Delta F / F$) after 10 μM Bay K 8644 treatment of control and *Tet1* cKO OPCs. Data are Means ± SD (n = 157 cells from three independent cultures in Ctrl group, n = 130 cells from three independent cultures in cKO group). ***, Two-tailed unpaired t test, t = 3.847, df = 285, p = 0.0001.

(e) The average rise in slope of Fluo4 intensity after Bay K 8644 addition in control and *Tet1* cKO OPCs. Data are Means ± SD (n = 157 cells from three independent cultures in Ctrl group, n = 130 cells from three independent cultures in cKO group). ***, Two-tailed unpaired separate variance estimation t test, t = 8.409, df = 280.3, p < 0.0001.

(f) Representative images of MBP / OLIG2 immunostaining of Bay K 8644-treated control and *Tet1* cKO OPCs. Scar bar, 100 μm.

(g) Percentage of MBP$^+$ cells among OLIG2$^+$ cells after 3 days of Bay K 8644 treatment in control

and *Tet1* cKO OPCs. Data are Means ± SEM (n = 3 of independent cultures each group). Two-tailed unpaired t test, Ctrl: *, t = -2.877, df = 4, p = 0.045; cKO: *, t = -3.655, df = 4, p = 0.022.

(h) Quantitative real-time PCR of *Cnp*, *Mbp*, and *Plp* after three days of Bay K 8644 treatment in control and *Tet1* cKO OPCs relative to vehicle treatment. Data are Means ± SEM of transcript levels after normalization from n=3 independent experiments each performed in triplicate. *Cnp*: One way ANOVA, $F_{(3,8)}$ = 1278, p < 0.0001; Ctrl vs. Ctrl+Bay K 8644: ***, q = 69.94, df = 8, p < 0.0001; cKO vs. cKO+Bay K 8644: *, q =5.594, df = 8, p = 0.0177. *Mbp*: One way ANOVA, $F_{(3,8)}$ = 1436, p < 0.0001; Ctrl vs. Ctrl+Bay K 8644: ***, q = 71.04, df = 8, p < 0.0001; cKO vs. cKO+Bay K 8644: ***, q =10.34, df =8, p = 0.0004. *Plp*: One way ANOVA, $F_{(3,8)}$ = 1530, p <0.0001; Ctrl vs. Ctrl+Bay K 8644: ***, q = 79.90, df = 8, p <0.0001; cKO vs. cKO+Bay K 8644: ***, q =14.61, df =8, p <0.0001.

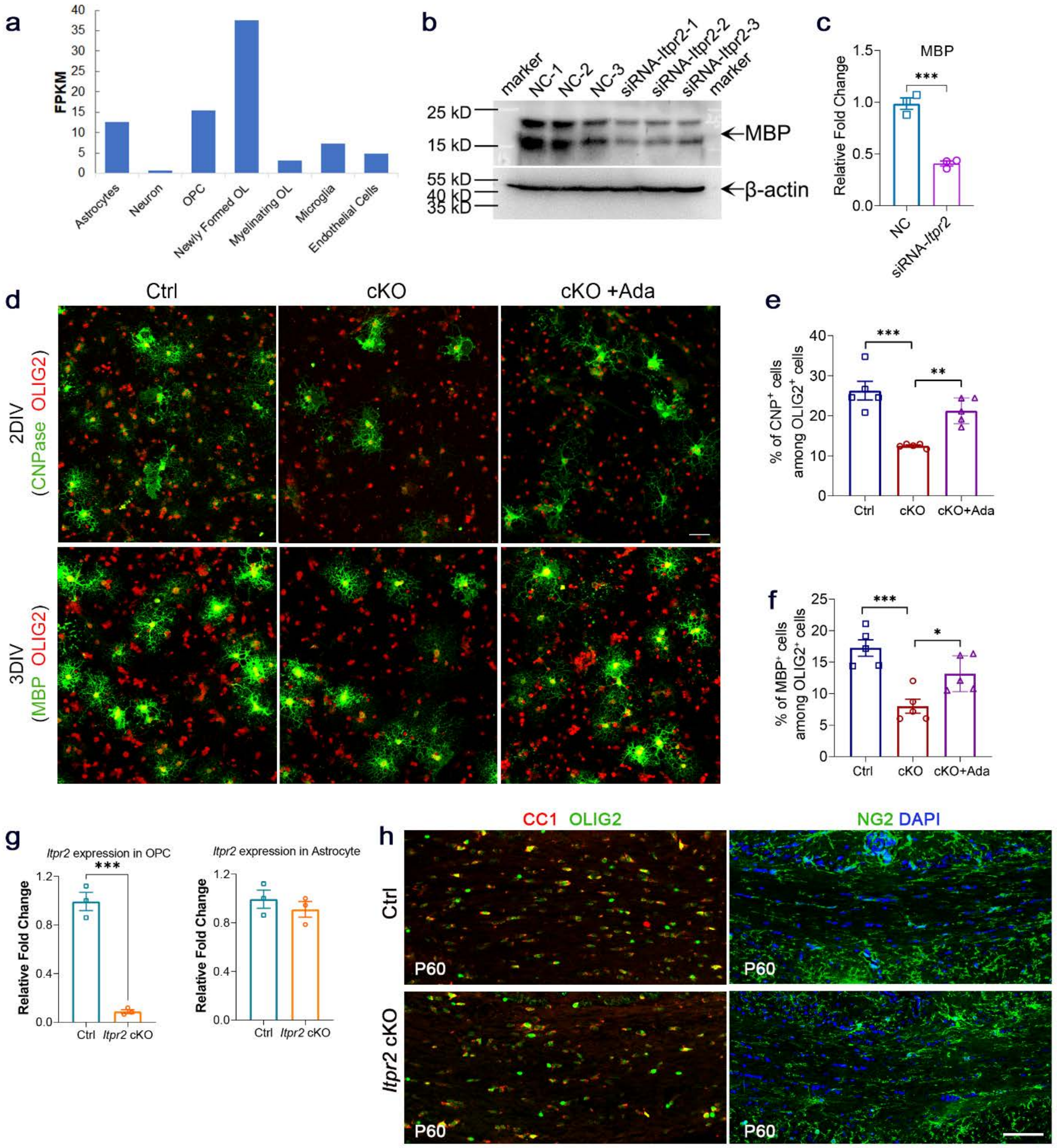

Supplementary Fig. 12 ITPR2, one of TET1-5hmC target genes, is required for proper OPC differentiation

(a) Column diagram shows the expression of ITPR2 in different neural cell types. The diagram is made from data in http://jiaqianwulab.org/resource.htm.

(b) Western blot assay for MBP showed decreased expression in siRNA-*Itpr2* transfected OPC cultures after three days in differentiation medium.

(c) Histogram shows MBP expression fold change measured by densitometry in siRNA-*Itpr2* group relative to control after normalization to β-actin levels. Data are Means ± SEM (n = 3 transfections). ***, Two-tailed unpaired t test, t = 9.388, df = 4, p = 0.000717.

(d) Representative images of CNPase, MBP and OLIG2 immunostaining in control, *Tet1* cKO and Ada-treated *Tet1* cKO OPCs at 2DIV and 3DIV. Scale bar, 50 μm.

(e) Percentage of CNP^+ cells among $OLIG2^+$ cells after 2 days of Ada treatment in *Tet1* cKO OPCs. Data are Means ± SEM (n = 4 of independent cultures each group). One way ANOVA, $F_{(2,12)}$ = 19.422, p = 0.000173. Tukey's multiple comparisons test, Ctrl vs. cKO: ***, q = 8.708, df =

12, p = 0.0001, Ctrl vs. cKO+Ada: q = 3.169, df = 12, p = 0.1042, cKO vs. cKO+Ada: **, q = 5.539, df = 12, p = 0.0054.

(f) Percentage of MBP^+ cells among $OLIG2^+$ cells after 3 days of Ada treatment in *Tet1* cKO OPCs. Data are Means ± SEM (n = 5 of independent cultures each group). One way ANOVA, $F_{(2,12)}$ = 14.058, p = 0.000717. Tukey's multiple comparisons test, Ctrl vs. cKO: ***, q = 7.485, df = 12, p = 0.0005; Ctrl vs. cKO+Ada: q = 3.306, df = 12, p = 0.0886, cKO vs. cKO+Ada: *, q = 4.180, df = 12, p = 0.0300.

(g) qRT-PCR assay tests the mRNA expression of *Itpr2* in OPCs and astrocytes purified from control and *Itpr2* cKO mice. Data are presented as Mean ± SEM of transcript levels after normalization from n=3 independent experiments each performed in triplicate. Two-tailed unpaired t test, OPC: ***, t = 11.794, df = 4, p = 0.000296; Astrocyte: t = 0.852, df = 4, p = 0.442.

(h) Representative immunostaining of CC1 / OLIG2 and NG2 in corpus callosum of P60 mice. Scale bar, 50 μm.

Supplementary Table1. Summary of 5hmC peak region, primer information and results for BisulPlus™ Loci 5hmC-qPCR assay in certain genes.

	Gene Name (Chr #)	5hmC peak region	Hydroxymethylated primers	Un-hydroxymethylated primers	Relative 5hmC abundance	p value
OPC associated genes	*Cspg4* (Chr9)	56861264-56862568	TTTAGATAGAAAAGTATTTGTCGGG<br>TTTAGATAGAAAAGTATTTGTCGGG-3	TTAGATAGAAAAGTATTTGTTGGGG<br>AATTTCCCTTAACTTAAATCAACCA	2.576	0.0148
	Slc22a3 (Chr17)	12498956-12503627	ATTATATCGACGAAGAGGAAGGC<br>CAAACACTAAAAAAAACGAACGAAT	TCCAGACTCTCCCTGCTTAAC<br>TCTTCTCTCCTTCTGCCACTG	41.810	<0.0001
	Wnt10a (Chr1)	74792174-74793286	TCCAGACTCTCCCTGCTTAAC<br>TCTTCTCTCCTTCTGCCACTG	TTATATTGATGAAGAGGAAGGTGAAG<br>CCAAACACTAAAAAAAACAAACAAAT	2.011	<0.0001
iOL associated genes	*Kndc1* (Chr7)	139893099-139894079	TAGGTTGAGATGTATTAGTTGTGGC<br>AATACTTTCCTCTAATTAACGCTCA	GAGATGTATTAGTTGTGGTGTGT<br>AATACTTTCCTCTAATTAACACTCA	17.010	0.0322
	Olfr279 (Chr15)	98496136-98496853	TTAGGAAAATAAATAATTTTCGAGA<br>AAAAACCAAAACATCTACCCTACG	TTAGGAAAATAAATAATTTTTGAGA<br>AAAAAACCAAAACATCTACCCTACA	1.686	0.0187
	Zdhhc12 (Chr2)	30093033-30093493	GAGTAGGGTAATTATTAACGCGAAG<br>ACCCTAAAAACTATAACTCCGATCC	GGAGTAGGGTAATTATTAATGTGAAG<br>TCCAACCCTAAAAACTATAACTCCA	1.179	0.0412
mOL associated genes	*Mag* (Chr7)	30900452-30901587	TAATGGTAATTAGGATGGTAAAGGC<br>CCCTAAAAACTCCTAATCTCGTTCT	TGGTAATTAGGATGGTAAAGGTGAT<br>CCCTAAAAACTCCTAATCTCATTCT	3.170	<0.0001
	Elovl7 (Chr13)	108215666-108216506	GGTTTCGAATTTTTTGTGTAGTTTC<br>CTAACAAACATAAAACCTCCGATTT	TTTGAATTTTTTGTGTAGTTTTGGAT<br>CTAACAAACATAAAACCTCCAATTT	9.007	0.0383
	Arl2 (Chr19)	6139651-6140428	TATTTTGTTAGGTAGGTTTTCGAAG<br>ACTAAAACCCAAATATAAACGCTTA	TATTTTGTTAGGTAGGTTTTTGAAG<br>ACTAAAACCCAAATATAAACACTTA	1.641	0.0346

	Cdc42ep2 (Chr19)	5924966-5926196	AATCGAGGGAAATTTTTTATTGC<br>CAACCTATACCTAAAACCTCGAATT	AAATTGAGGGAAATTTTTTATTGTGT<br>CAACCTATACCTAAAACCTCAAATT	3.086	0.0078
Negative regulator	*Zfp28* (Chr19)	6387100-6387562	AATCGAGGGAAATTTTTTATTGC<br>CAACCTATACCTAAAACCTCGAATT	AAATTGAGGGAAATTTTTTATTGTGT<br>CAACCTATACCTAAAACCTCAAATT	0.8052	<0.0001
	Ngf (Chr3)	102471240-102471702	AGTTTAGAGGGTAGTGTAGTCGTTT<br>GAAAACCGAATATAACCCGTAAC	AGTTTAGAGGGTAGTGTAGTTGTTT<br>ACAAACAAAAACCAAATATAACCCA	0.1127	0.0489

Supplementary Table 2. Transcriptome changes in OPCs from *Tet1* cKO mice

Upregualted gene	Log2 (cKO/OPC)	Downregulated gene	Log2 (cKO/OPC)
Ifi202b	9.145461947	Clca1	-7.175680633
Tlr1	7.216005351	Ttr	-6.751171351
Ccl6	6.581469776	Cdh5	-6.418979102
Chrna10	5.859272075	Gldn	-5.926284196
Nts	5.680285631	Slc14a1	-5.506635508
Rprml	5.460092219	Serpina3n	-5.363509847
Egr4	5.311048922	Cyp3a13	-5.303327196
4930539E08Rik	5.191022921	Klk6	-5.200460001
Sp140	5.138193072	Gdnf	-5.165446393
Cd74	5.115591761	Cd34	-4.980227037
Clec4n	5.01833284	Mmp13	-4.871453224
Pf4	4.851203631	Gprc5c	-4.854812179
Olfr287	4.807492251	Fam19a1	-4.711317976
Ankrd63	4.691102151	Tmem196	-4.710234354
Gpr88	4.565407171	Eps8l1	-4.644114901
Rpe65	4.50694568	Ppl	-4.626566012
Gabrd	4.48625855	Ptgfr	-4.600421631
Ipcef1	4.302236827	Alox5	-4.592833791
Fcgr2b	4.301501803	Slc5a7	-4.576190491
Rasgrp1	4.280273614	Nek5	-4.541786201
Ccl7	4.232637713	Dmp1	-4.482922315
Drd2	4.19944924	Prl2c2	-4.471212499
Hist1h2ao	4.129084596	Tnn	-4.470936612
Penk	4.113720446	Epcam	-4.384141741
Cxcl3	4.083162402	Akap14	-4.376001719
Acpp	4.067836595	Mobp	-4.367261996
Gjb6	4.038321878	Gm6588	-4.338490814
Tal1	3.967374033	Tmem132c	-4.278063047
Rxrg	3.90493484	Mag	-4.229839775
Ddn	3.877639332	Akr1b8	-4.22760776
Il2rg	3.828184135	Gjc2	-4.213118585
Aldh1a1	3.77992922	Thsd4	-4.206536565
Alox5ap	3.771105764	Cldn11	-4.201819655
Ucp2	3.719785286	Lingo2	-4.167865308
Mx1	3.682453465	Slc24a2	-4.122341697
Prdm8	3.682057339	Syne3	-4.054357343
Wfdc17	3.668146027	Tcea3	-4.038440154
Fpr2	3.647745314	Tmprss5	-4.033205429
Matn4	3.639746697	Gpr149	-3.987163254
Sstr3	3.625836727	Slc9a2	-3.982179637
Vax2	3.624722319	Foxd3	-3.963006175
Cd33	3.620399911	Nipal1	-3.951699209
Saa3	3.614482374	Tktl1	-3.945976033
Dclk3	3.614328493	Speer4b	-3.929919951
Hist1h2an	3.566203981	Lef1	-3.925884721
Dhrs7c	3.54404446	Ndst4	-3.913697554
Kcnh3	3.533685654	Efhb	-3.907454539
Ccl3	3.524430921	Prima1	-3.895949966
Hapln1	3.524312164	A930009A15Rik	-3.876244044
Tmem210	3.517017083	Mbp	-3.83788806
Klk13	3.499657105	Lpar1	-3.807297249
Pou6f2	3.499652366	Arhgap28	-3.804787829
Nrsn2	3.465196646	Il22ra1	-3.799150889
Camk2a	3.447694419	Sytl2	-3.793151505
Helt	3.422689413	Ano1	-3.791471429

Cacng3	3.416599066	Ifi205	-3.789019082
Odf3l1	3.397542268	Spag16	-3.783930517
Pla2g7	3.392131877	Enpp6	-3.775748763
Slc6a7	3.377622996	Tmprss3	-3.775405949
Ebf2	3.36749516	Birc7	-3.758199949
Mal2	3.367493463	Rec8	-3.757412912
Il1f9	3.357380671	Adh7	-3.753535519
Camkv	3.320219134	Pappa2	-3.752998563
Susd3	3.298071882	Gprc5a	-3.715098176
Npr3	3.296655547	Ppp1r36	-3.688030186
Nrgn	3.279747676	Gpr55	-3.672814244
Lcn2	3.26996332	Tmem184a	-3.671877323
Serpinb6b	3.267353925	Egfl6	-3.667046112
Hal	3.262207906	Nr1h4	-3.654957232
Slc39a12	3.25498555	Nostrin	-3.654947293
Ccr7	3.250056197	Fndc3c1	-3.654740402
Rab37	3.225434877	Tex22	-3.647553772
Gjd3	3.212815059	Tmem125	-3.632111551
Rgs14	3.197068637	Zfp92	-3.609079751
Lao1	3.194894621	Plp1	-3.606409287
Hpcal4	3.184335316	Kcne4	-3.58134193
Gm5796	3.181971517	Sprr1a	-3.555737305
Pou3f1	3.179737714	Fam110c	-3.550938659
Grem2	3.17488368	Tmem163	-3.54313798
Htr1a	3.173771425	Ccl28	-3.528091568
Atp6v0d2	3.172856559	Fhad1	-3.501407616
Asb16	3.172853144	Trabd2b	-3.484577166
Clic5	3.148546678	Ccdc162	-3.479230728
Ppp1r3g	3.141959174	Trf	-3.4720488
Hmgcs2	3.134186244	Ccdc60	-3.471533956
Sowaha	3.122734184	Slc15a1	-3.468361216
Slc25a34	3.120880394	Col8a1	-3.457725791
Nxnl2	3.097680554	Igfals	-3.442750176
Cd300ld	3.095274999	Akr1c21	-3.429767736
Slc35f4	3.088044553	Prl2c3	-3.424838901
F13a1	3.08161526	Tfpi2	-3.423232885
Tpo	3.069685574	Kndc1	-3.421942274
Gngt2	3.067836848	Spp1	-3.402946935
C130060K24Rik	3.045003338	Snai2	-3.392946377
Npy2r	3.039712243	Myrf	-3.379653316
Foxc1	3.037269013	Tmem159	-3.379510798
Apoe	3.023137061	Ret	-3.375481099
Scn10a	3.018074335	Cd59a	-3.367857367
Tlr6	3.015470859	Slc17a8	-3.349811351
Baiap2l1	3.011716802	Slit2	-3.347995862
Fam43b	3.011524897	Apod	-3.345802811
Gbp10	2.9906383	Arntl2	-3.340599725
Synpo2l	2.98887623	Kctd19	-3.334151869
Samsn1	2.982233376	Rbp3	-3.323666317
Gm1604b	2.962660875	Ifitm1	-3.323664156
Epx	2.957136201	Veph1	-3.314188028
Egr1	2.94532322	4933411K16Rik	-3.313098932
Unc13c	2.929221172	Angpt4	-3.313096375
Krt14	2.921874411	Gjc3	-3.3039095
Chrm3	2.910403595	Capn11	-3.292896716
Ifi44	2.904876113	Elovl3	-3.26964341
Glycam1	2.894862963	Hapln2	-3.237649121
Fgf8	2.8908934	Flt4	-3.237412715

Tmem71	2.890891022	Ephx4	-3.23567259
Actg2	2.890888277	Rab26	-3.229114704
Clec4e	2.851771824	Muc6	-3.229021478
Asb2	2.848519914	Nfe2l3	-3.215170174
Rph3a	2.848274692	Mylk2	-3.207133998
Sptssb	2.829434828	Piezo2	-3.202076206
Fxyd3	2.826853824	Flnc	-3.198930083
Dbh	2.826852417	Foxd1	-3.197115468
Egr3	2.789316245	Popdc2	-3.185682595
Ccr2	2.782025605	Gata3	-3.178420862
Gna15	2.763975492	Rab3b	-3.169551229
Olfr1392	2.749659795	Lypd6b	-3.162812728
Fcgr1	2.743916856	Sgk2	-3.139728962
Bspry	2.737699477	Tmem204	-3.139469429
Hcrtr1	2.731362212	Sytl5	-3.13862172
Sycp1	2.729798765	Ttc21a	-3.132409053
Rasgrf1	2.722233844	S1pr5	-3.131770765
Itk	2.707267509	Mylk3	-3.126303602
Ccl5	2.695346039	Klrg1	-3.120745167
Wnt1	2.689369616	Lpcat2	-3.11982106
Zpbp2	2.682650093	Col12a1	-3.11980791
Hs3st4	2.676181324	Esr2	-3.113659237
Kirrel2	2.673021238	Thbs2	-3.113283076
Fetub	2.671048291	Rhox5	-3.110128789
Tacr3	2.665205855	Gbp6	-3.107478885
Mettl11b	2.652699433	Col8a2	-3.105792327
Selplg	2.652042588	Mgst2	-3.099381425
Sla	2.640397466	Ermn	-3.09863447
Lrrc25	2.629597422	Cntn3	-3.097906489
Pax4	2.61527402	Cdk18	-3.08812629
Dpep2	2.612108377	Myo1d	-3.081809351
Ncf4	2.612071378	Itgb3	-3.074815982
Prss41	2.611974462	Cabp4	-3.067923462
Egr2	2.593415631	Treh	-3.063869448
Slc6a11	2.586064353	Cdh7	-3.063863712
Hp	2.583408233	Pm20d1	-3.060417267
Gm9992	2.57686017	Myo18b	-3.058316185
Fcrlb	2.576857991	Il12rb1	-3.042707814
Isl2	2.575978175	Ahnak	-3.041502647
Mrc1	2.553214439	Pax9	-3.038440032
Ppp1r1b	2.546294082	Tm4sf1	-3.03257173
Klhl38	2.538076101	Slc9a3	-3.029859277
Itgax	2.538071814	Atp8b1	-3.017479885
Myadml2	2.526543348	4930467E23Rik	-3.016770324
Phyhip	2.526505285	Sbspon	-3.01484055
Pnoc	2.519624119	Crym	-3.007657143
Tnip3	2.496811138	Hes3	-3.006237329
Cpne6	2.487721969	Maats1	-2.993803752
Apoc1	2.482460867	Plec	-2.972073523
Nlrp3	2.482375024	Fa2h	-2.970129893
Plcb2	2.479950929	Tinagl1	-2.968898612
Tnfaip8l3	2.45667488	Cnbd2	-2.955503615
Acaa1b	2.456147792	Tspan2	-2.950561882
Epha8	2.437937828	Pth2r	-2.942668803
Hist1h2ad	2.4214208	Gucy2e	-2.935715874
Gm813	2.420148538	Chrna1	-2.935713754
Serpinb7	2.411817053	Casq1	-2.934365956
Igfbp4	2.397401279	Stx19	-2.929921385

C3	2.393535633	F2rl1	-2.918822682
Rasgef1a	2.382212459	Itga2	-2.918398422
Mpo	2.377621935	Mboat1	-2.907621089
Aldh1a7	2.375893101	F5	-2.897284828
Csf2rb	2.375468267	Ly75	-2.897195521
Apoc2	2.374629829	P2rx2	-2.891511532
Prr15l	2.367243667	C1qtnf3	-2.883405741
Slamf1	2.36724042	Sytl3	-2.883405009
Hnmt	2.364907435	Bpifc	-2.883402422
Icam5	2.363923295	Tmc5	-2.877502418
Clec4d	2.36023286	Fam47e	-2.869046927
Runx3	2.359848204	Hkdc1	-2.864233004
Ptger3	2.350586814	Sspo	-2.863290158
Ceacam16	2.330414339	Ostn	-2.863083839
Rnf43	2.316595405	Abcg3	-2.863076196
Cyp4f15	2.299747047	Cldn14	-2.859105775
Gstm1	2.296173148	Pla2g2c	-2.854610409
Gimap1	2.290044446	Nxf2	-2.854558202
Tmem221	2.290040795	Ankrd2	-2.854554669
Tfap2a	2.288246245	Abcc2	-2.854553308
Arhgap36	2.287024112	Mettl21c	-2.854551741
Oprm1	2.274267242	Rnf183	-2.854549972
Atoh7	2.27426484	Gm6583	-2.8496882
C1qtnf2	2.2742638	Pla2g10	-2.844806259
Cbln4	2.261812238	Cyp1a1	-2.843184857
Slc6a13	2.258171345	Tnnt2	-2.837511192
Golga7b	2.257938192	Parvb	-2.833312582
Cd300lf	2.256000672	Susd5	-2.833257275
Sh3tc1	2.247101278	Cd80	-2.8326462
Clec5a	2.244301921	Col4a6	-2.830832833
Ldlrad2	2.244294221	Ptk6	-2.828878283
Cd52	2.24065517	4930444G20Rik	-2.82887611
Gm5741	2.235150397	Mrgprf	-2.825118615
Kcnc4	2.234758631	Arhgef28	-2.805673561
Pld4	2.229844516	Slc6a20b	-2.783930626
Glp1r	2.206246342	Zfp951	-2.783789528
Tpd52l1	2.202967596	Col16a1	-2.781820599
Cnnm1	2.191852062	Ulbp1	-2.779247644
Gpr6	2.181434053	Mog	-2.776958546
Lrrc3b	2.174889441	Padi2	-2.766445927
Olfr1344	2.17488777	Otog	-2.764850925
Bsx	2.174880665	Atp10b	-2.7599242
Opn5	2.174880483	Nlrp1a	-2.758202492
Adprhl1	2.174690536	Crybb3	-2.752999713
A630001G21Rik	2.172851279	Dcdc2c	-2.736400346
Adra2a	2.169607846	Tfap2c	-2.734143503
Ankk1	2.148635843	Stra6	-2.73267254
Cd14	2.129969597	Jph2	-2.731582256
Ranbp3l	2.127672002	Col18a1	-2.729552948
Prok2	2.125879673	Col20a1	-2.726151176
Slc27a2	2.12317788	Vmn2r29	-2.719730398
F10	2.123039311	Ahrr	-2.713312753
Efhc2	2.109524266	Clic6	-2.713074643
Scgb1b30	2.097683986	Il1r1	-2.710692907
Eva1a	2.090768055	Lax1	-2.709962535
Cpne5	2.088358583	Prelp	-2.705196221
Crhr1	2.086137965	Opalin	-2.704839189
Lck	2.079785612	Unc5b	-2.70383542

Srgn	2.077624654	Tmem132d	-2.70214402
Cldn10	2.077289399	Btbd16	-2.699520166
Zmat4	2.073845709	Apol6	-2.698893458
Palmd	2.066553722	Nipal4	-2.696521005
H2-T10	2.066439047	Rassf10	-2.693612439
Cbr3	2.064841678	Exph5	-2.692249462
Rs1	2.062784343	Otop1	-2.69104934
Lrrn4	2.062480636	Klhl31	-2.691045742
Galnt9	2.062155051	Sftpb	-2.688034648
Pla2g4e	2.053967799	Vsx2	-2.688032062
Proser2	2.052664779	Orm2	-2.688031961
Scel	2.046251212	Gpr18	-2.688031577
Adam1b	2.045001992	Psmb11	-2.688031184
Nos2	2.042508087	Olfr543	-2.686885337
Ccr5	2.041639592	Sema3g	-2.686804646
Fgf5	2.039859848	Barx2	-2.685675788
Fosb	2.038199414	Serpine1	-2.684263335
Naip5	2.03031144	Sgms2	-2.680551682
Mroh7	2.023056056	Styxl1	-2.677093006
Fgf9	2.022624459	AW551984	-2.676160369
Nell1	2.020615739	Tex26	-2.671876686
Aadat	2.005981146	Klk7	-2.67187408
Il21r	2.000746585	Arhgef5	-2.662459514
Fabp4	2.000745017	Mnda	-2.662322263
Me3	1.999290934	Cidea	-2.66219344
Kcnj4	1.998580941	Morc2b	-2.659999591
Cyth4	1.997727571	Enpp2	-2.654274874
Klk10	1.98900708	Scara3	-2.652453353
Car3	1.987492606	Neil3	-2.652079052
Clec3b	1.985454781	Col23a1	-2.649634541
Cxcl2	1.9853916	Mmp3	-2.645779675
Spock3	1.984539093	Scgb3a1	-2.643757517
Wnt4	1.980737001	Angptl2	-2.634838577
Baalc	1.968173112	Glp2r	-2.629091351
Tmem255b	1.967448713	Medag	-2.620655995
Lta	1.964463164	Pappa	-2.615139645
Rasal3	1.964336329	S100b	-2.611985467
Itih4	1.962658703	Pmp2	-2.60523196
Mybpc1	1.959312856	Klk8	-2.601657702
Fxyd1	1.95499176	Bcas1	-2.599868263
Rbp4	1.953112694	Kif14	-2.592344952
Htr7	1.952337564	Tnni1	-2.590764021
Cd163	1.952205562	Ddc	-2.588994066
Lyz2	1.944153155	Mns1	-2.587217995
BC049715	1.94340725	Bdkrb2	-2.585082403
Myh8	1.939170207	Il1r2	-2.579437637
Rgs11	1.934030825	Elovl7	-2.576357642
Wscd2	1.931181458	Igbp1b	-2.576189151
Smco3	1.929056116	Prkg1	-2.56250478
Tbx2	1.928321424	Agtr1a	-2.56064377
Atp6v1e2	1.924694891	Afap1l2	-2.556225453
Fxyd7	1.923814056	Pof1b	-2.555734504
Ifit1	1.923786225	Sox7	-2.552312404
Ripk4	1.921866961	Enpp3	-2.546786811
Ttpa	1.92086168	Cdsn	-2.546352322
Nefh	1.914478817	Spata18	-2.54604539
Prkcg	1.913942599	Adamts16	-2.54604404
Adora2a	1.910819931	Pla2r1	-2.545719479

Klhl6	1.908955572	Xirp1	-2.54283496
Slc47a2	1.890891636	Rab44	-2.541086088
Pde6b	1.890891071	Kif19a	-2.537791865
Plscr5	1.890889996	Clec2d	-2.535036257
Gpr132	1.890888883	Heyl	-2.529969994
Fam83b	1.890886977	Nov	-2.522674414
Tmem158	1.890522905	Rasgef1b	-2.522088591
Itpka	1.882428668	Adamts4	-2.522029462
Tnfrsf14	1.875009495	Vwf	-2.517800969
Irgc1	1.875000008	Hfm1	-2.517686609
Oprk1	1.874996629	Otx2	-2.517463074
Siglec1	1.868506648	Slc12a3	-2.517313561
Rbpjl	1.868127185	Tph2	-2.515867931
Lpo	1.86666659	Tmem88b	-2.51490219
Pdzd3	1.865294764	Tfap2e	-2.506532115
Rasl11a	1.860996841	Asap3	-2.504956859
Gstm6	1.855005728	Msx1	-2.502201883
Wnt2	1.840600734	Fhl2	-2.49727203
Prmt8	1.836506356	Olfr920	-2.495679133
Klf2	1.83599421	Ddr2	-2.495425543
Orm1	1.82685391	Car9	-2.488124502
Kcnv1	1.826050469	Met	-2.484523958
Sncb	1.825219554	Efcab1	-2.47639853
Sp100	1.822324603	Mmel1	-2.474695118
Trhr	1.819175723	Klk11	-2.474694672
4930502E18Rik	1.813691087	P2rx7	-2.46870075
Acot11	1.810814272	Thbd	-2.468060014
Lyz1	1.806224041	Twist2	-2.466310679
Osm	1.80486203	Fbxl13	-2.462591829
Csf1r	1.804762473	Npsr1	-2.462000319
Slamf7	1.800660068	Cd300lg	-2.461999915
Ntsr2	1.791456082	Gm6592	-2.461999914
Gstt1	1.790613992	1700034J05Rik	-2.461999284
Cdh17	1.787740889	St18	-2.458659014
Rbfox3	1.787381531	Ifnlr1	-2.455613591
Vav1	1.785924963	Opcml	-2.454156853
Rasal1	1.77895843	Fam216b	-2.453364603
Mmp9	1.777254959	Gpa33	-2.451400755
Slc25a18	1.772624655	Fgf7	-2.451031836
Pla2g5	1.770303426	Car10	-2.449385593
Hcls1	1.766016367	Mylk4	-2.44919867
Camk2n1	1.760265449	Guca2a	-2.449197173
Fgf3	1.759850176	Mansc4	-2.449162796
Nr5a1	1.759849405	Trpv4	-2.442582257
Cd4	1.759845381	Sulf1	-2.442309983
Barx1	1.759840763	Hhip	-2.441791364
Gm5105	1.754625354	Chst8	-2.441041568
Oaf	1.749693923	Gimap8	-2.436275876
Wfdc6a	1.7496565	Prss45	-2.436273914
Npw	1.749655793	Olfr279	-2.436270028
Nkx1-2	1.749655205	Mki67	-2.436192099
Neurog2	1.747313824	Ror1	-2.430831962
Kcnk12	1.746827597	Ooep	-2.429766644
Mmp12	1.746638944	Tmem74	-2.428967738
Epas1	1.746101131	Sntg1	-2.427594591
Cds1	1.744015457	Gsc	-2.426573447
Bin2	1.742851263	Naalad2	-2.426012333
Dhrs2	1.736869372	Trim12a	-2.421021002

Arc	1.73537535	Eid3	-2.419471434
C1ra	1.733276042	Ngf	-2.417588565
Dennd2d	1.730480439	Igfbp6	-2.417240275
Efna3	1.726022409	Olfr545	-2.416672576
Mchr1	1.722759727	Armc3	-2.415078994
Sidt1	1.720537806	Igf2	-2.413261658
Hoxc9	1.716541228	Igf2bp1	-2.412715438
Itgam	1.714022729	Loxl2	-2.412347927
Usp44	1.700965214	Ldb3	-2.412162196
Ms4a6b	1.700613116	Adora3	-2.410077278
Ccl22	1.697104777	Itga11	-2.40395764
Lrmp	1.689992887	Tek	-2.401859093
Folh1	1.688210791	Dusp26	-2.401222646
Ctss	1.687420652	Lhx9	-2.398789583
BC024139	1.685819551	Ddx4	-2.398647497
Pgr	1.685252372	Ngfr	-2.387633155
Tifa	1.683797199	Slc2a12	-2.380378707
Gda	1.682347828	Slc10a6	-2.377894442
Ccl9	1.660884585	Cox4i2	-2.375280046
Tlcd1	1.659541884	Nfasc	-2.37441999
Cacna1i	1.659367821	Atp8b3	-2.374289063
Sp7	1.655026667	Nek11	-2.373731169
Caln1	1.65433727	Syt15	-2.370866173
Gpr31b	1.650055819	Mlkl	-2.370104398
Gas2l2	1.648336863	Mgp	-2.366998462
Plcd4	1.640396412	Slc9b1	-2.362616423
Cst3	1.638959088	Cyp26b1	-2.357203422
Aoah	1.638896467	Bmp6	-2.353524602
Ccl4	1.63738143	Aldh8a1	-2.348018866
Trim75	1.637379221	Grid2	-2.346248711
Inpp5j	1.630278618	Aspm	-2.343554644
9930111J21Rik1	1.63006511	Morn5	-2.343367131
Cdkl1	1.629356295	Ndrg1	-2.337389455
Ncf1	1.627973057	Perp	-2.335578745
Nxnl1	1.626001988	St14	-2.333654558
Gpr12	1.624559107	Cd244	-2.333201225
Phkg1	1.624262735	Clrn1	-2.328735557
Gbp5	1.613923113	Timp1	-2.327491379
Fam159a	1.613576826	Rho	-2.322063996
Actc1	1.613168092	Msln	-2.320153816
Ly9	1.605553898	Gsta2	-2.320152352
Syt12	1.605231208	Cped1	-2.319579994
Glo1	1.604700314	Tnxb	-2.31697889
Cytip	1.601660311	Fgf18	-2.315382009
Lars2	1.59939962	Clhc1	-2.309474552
Trim29	1.599311991	Prune2	-2.307741182
Ripply2	1.597956019	Pxdc1	-2.305770346
Baiap2l2	1.595228421	Ypel2	-2.30213091
Ptpn18	1.588075988	Syne2	-2.301134484
A830018L16Rik	1.579582688	Il13ra1	-2.297451569
Sowahb	1.576861804	Clmn	-2.294935197
Fam167b	1.576858413	Tmem40	-2.287635832
S100a8	1.576857895	Ddo	-2.280388215
Apol9b	1.576856122	Lrrc9	-2.276624366
Vmn1r58	1.576159574	Trh	-2.275724677
Zfp345	1.575499022	Hist1h3b	-2.274248829
Scn7a	1.575496105	Sstr5	-2.269646519
Clec4a1	1.572052879	Lhfpl5	-2.269646223

Rnf151	1.571767223	Iyd	-2.269643757
Apobec1	1.570703901	Cdh12	-2.266087307
Sec14l3	1.568964594	Ptpre	-2.263252821
Dll3	1.568476853	Rhpn2	-2.261980917
Ism1	1.56795832	Pmaip1	-2.259960326
Abi3	1.559807241	Ptchd4	-2.259194051
Nab2	1.559149507	Clnk	-2.256839541
Lypd6	1.557355464	AU018091	-2.256839231
Npas4	1.553890457	Angpt2	-2.254988435
2010300C02Rik	1.551034956	Tm4sf4	-2.243919082
Rd3	1.551001103	Pkp1	-2.243917594
Agmo	1.542525617	1700047I17Rik2	-2.243914866
Bdh2	1.540151719	Hsd3b3	-2.24391393
Tmco5	1.538073646	Osr1	-2.242483839
Slc47a1	1.53807175	Klhl4	-2.24171678
Sp110	1.537996453	Mcam	-2.23602914
Myo1g	1.537878665	Clgn	-2.234759447
Lrrc6	1.537739679	Uchl4	-2.227785365
C1rl	1.53739017	Adrb3	-2.227784219
Cabp7	1.537388695	Gpr35	-2.227600577
Gpr61	1.534051234	Has1	-2.221902542
Syndig1	1.530387899	Lrtm1	-2.221877011
Ckmt1	1.524764631	Hdc	-2.22076784
Cwc22	1.524011943	Tec	-2.220208527
Fmo1	1.522649623	Hgf	-2.219206827
Csf2rb2	1.520373832	Mrgprb5	-2.217723014
Ung	1.519498213	C7	-2.211194639
Tnf	1.518569986	Ptx3	-2.210713725
Ccdc85a	1.513521874	Bpifb5	-2.209135519
Flywch2	1.513127417	Gsc2	-2.209129956
Jph4	1.51223961	Tfap2b	-2.209129914
Cpne4	1.511297141	Abhd14b	-2.208869022
Kcne1l	1.509985777	Prom2	-2.206634789
Npas2	1.50994897	Sertm1	-2.205098963
Camk2b	1.509635942	Rnasel	-2.204095997
Cdk5r2	1.508936037	Nr1i2	-2.201517038
Il11ra1	1.505736211	Pla1a	-2.201516187
Ybx2	1.50418382	Matn1	-2.201367276
Nfam1	1.500662347	Tnc	-2.201354196
Pcp4	1.500129212	Slc34a3	-2.198963491
Ifitm6	1.499657059	Plcg2	-2.198264119
Gstm3	1.499655631	Stk32b	-2.195554361
T	1.499654133	Cenpe	-2.195079195
Galnt18	1.498593074	Entpd4	-2.194641604
Sfrp5	1.492046641	4931429L15Rik	-2.18615992
Plbd1	1.491795482	Sema5a	-2.185964793
Pak6	1.488237093	Tsx	-2.183588465
Nrarp	1.4874017	Mal	-2.182229662
Dleu7	1.487195587	Trim36	-2.181335418
Hddc3	1.48523812	Ccdc114	-2.181068546
Paqr7	1.484848187	Rfpl4	-2.178425628
Mesp2	1.481951896	Gckr	-2.178424447
Doc2b	1.481209204	Neurog1	-2.1784215
Vstm2l	1.481153445	Ccdc158	-2.178420047
Prrt1	1.477187455	C130026I21Rik	-2.178419029
Fam163b	1.476903821	Pamr1	-2.177200457
Ntsr1	1.476383427	Tppp	-2.17439248
Phospho1	1.473258592	Fam187a	-2.170643543

Fmnl1	1.467193706	Arsj	-2.164956477
Ms4a6d	1.466914607	Crabp2	-2.162807695
Slc5a9	1.466089227	Ceacam1	-2.162667997
Rac2	1.463684085	Mob3b	-2.161066126
Rlbp1	1.458334957	Dkk3	-2.160323344
Cd38	1.45420244	Kif11	-2.159609771
Th	1.45180168	Prokr1	-2.1580233
Hist2h2ac	1.451258358	Col17a1	-2.157843086
Ajap1	1.450715876	1810024B03Rik	-2.157839231
Fcgr4	1.445001762	Ctse	-2.157838799
Col4a4	1.442755214	Cel	-2.155655291
Tox2	1.440221662	Myof	-2.154431061
Neurod6	1.436736174	Fn1	-2.15261974
Nlrp10	1.436735948	Grm6	-2.147046471
Cdhr1	1.436659106	Tgm1	-2.143580457
Tmem72	1.434245029	Gpr152	-2.141792551
Cdhr3	1.433318434	Dydc2	-2.141789801
Kremen2	1.431680927	Adamtsl4	-2.141533139
Tnnt3	1.428706738	E030018B13Rik	-2.137048841
Foxh1	1.426377922	Plxnb3	-2.133965449
Slc24a4	1.426218048	Nfatc4	-2.131835844
Samd1	1.425227188	Cenpf	-2.131263749
Rasgrp2	1.423514253	Esam	-2.130338291
Ccnb1ip1	1.419613462	Ccdc150	-2.128976948
Agtr2	1.41961107	Myl2	-2.127794597
Sp6	1.418788477	Capn3	-2.12172048
Gfra4	1.415683694	Itpr2	-2.121243506
Rhcg	1.412756477	Col2a1	-2.120784183
Lyn	1.409504227	Olfr1393	-2.120742152
Slc12a5	1.409233601	Eps8l2	-2.118685588
Crip1	1.409022276	Gm7173	-2.113923727
Slco1a4	1.406091277	Adssl1	-2.113896972
Was	1.406091042	4930590J08Rik	-2.113656982
5830473C10Rik	1.405672688	Tssk3	-2.113656389
Acsbg1	1.405575749	Pparg	-2.107107786
Rasl10b	1.405520555	Klf17	-2.106540445
Gls2	1.40305438	Trim43a	-2.106153868
Hmox1	1.400613971	A3galt2	-2.105919035
Psd4	1.399148052	Abca13	-2.103070368
Acaa2	1.399077292	Iqgap3	-2.101289616
Bend6	1.399031345	Hist1h4n	-2.096824799
H2-Ke6	1.397670721	St6galnac2	-2.095598109
Stc2	1.397214663	Gpr37	-2.094856922
Cnr2	1.396537014	Itga3	-2.094507432
Mlxipl	1.392686836	Pde1c	-2.093596126
Gdf10	1.392686809	Tnni3	-2.092127241
Dmkn	1.389825303	Map3k6	-2.091019462
Gpr22	1.388933693	Riiad1	-2.088741414
Hpdl	1.387847274	Itgb7	-2.087764931
Sv2c	1.387591467	Slc7a14	-2.08752095
Slfn2	1.386362149	Mdh1b	-2.086777317
Foxd2	1.383573659	Pkd2l1	-2.079040319
Lrfn5	1.38319776	Chrna5	-2.076883393
Gabra5	1.376890823	Ptprd	-2.070889903
Scn1b	1.376278823	Itgb4	-2.069486819
Hif3a	1.376065355	Fbxo32	-2.065908119
Vsig8	1.374454104	Dsc2	-2.063438275
Satb2	1.37182617	Plk5	-2.063434344

Fgf1	1.371780402	Ifitm10	-2.059876756
Chst1	1.370851302	Bgn	-2.059079757
Fcrls	1.36924072	Zbtb42	-2.058541059
Fcer1g	1.368810954	Endou	-2.055439933
Wnt16	1.367244826	Sall4	-2.055437914
Lgals7	1.367243567	Wfdc2	-2.054975537
Ckmt2	1.367242918	Grem1	-2.052737039
Lgals12	1.367242217	Gpr165	-2.051197008
Cdhr5	1.367241579	Col9a1	-2.050298672
Otof	1.367240412	Arhgef19	-2.047805971
Defb42	1.367239362	Fbln5	-2.045091178
Trim63	1.367239155	Igsf9b	-2.043677369
H2-Aa	1.36723852	Ugt8a	-2.040583499
4933409G03Rik	1.367238307	Ank3	-2.037285804
Bmx	1.367238137	Gm5868	-2.036059409
Hoxa7	1.367237866	Kif27	-2.035742573
Ngp	1.367237554	Scn5a	-2.033126043
Metrnl	1.365962146	Slc23a3	-2.031886477
Syt5	1.36531996	Speer3	-2.03188522
Pde4a	1.365308889	Cnga3	-2.029863008
Gbp3	1.362823057	Slc17a6	-2.029860588
Dlk2	1.358243809	Btbd8	-2.026079455
Ryr3	1.358206151	Pif1	-2.025814457
Asb5	1.356765618	Clec2g	-2.0256041
Fxyd5	1.356178835	Dpcr1	-2.024348947
Drd4	1.356101668	Arsi	-2.02076956
Rhoq	1.355995795	Trim72	-2.019741688
Cebpa	1.355329099	Rinl	-2.019217386
Hist1h2ah	1.355203179	Sox18	-2.016775655
Nupr1l	1.352940124	Tph1	-2.016774865
Kcnk3	1.352351512	Fsd2	-2.01677263
Dio3	1.351667946	Htr1f	-2.012554446
Alpk3	1.350711257	B3galt5	-2.008698101
Gfpt2	1.350356087	Smim6	-2.008146466
Gck	1.3495651	Ass1	-2.00622182
Gulp1	1.34781762	Cnpy1	-2.00545526
Fbxo2	1.346733198	Neu4	-2.005107503
Tmem37	1.344158526	Gm128	-2.004546687
Mdga1	1.341631742	Aldh3a1	-2.001692474
1700023F06Rik	1.341268542	Gnb3	-2.001502248
Sox9	1.340884758	Amdhd1	-2.001497494
Ttc34	1.339338896	Nsun7	-2.000654456
Dnm1	1.338909602	Entpd2	-1.999953388
Clybl	1.338591537	Erbb4	-1.997303872
Mt1	1.33517806	Gm6904	-1.993803588
Spta1	1.334772721	Bfsp2	-1.992616447
Myh7	1.334007174	Kif15	-1.98916442
Mtfp1	1.333991617	Tmem151a	-1.988946693
Ctf1	1.332211159	Sfrp4	-1.987689436
Xlr4b	1.331543924	Kbtbd12	-1.9860639
Cxcl12	1.330643944	Bex6	-1.986063528
Cyp27b1	1.328967129	1600014C23Rik	-1.986061794
Tgm3	1.326633047	Gsdma	-1.986060456
Ptges2	1.32449076	Zdhhc22	-1.982651981
Wif1	1.323249615	Vcan	-1.982533616
Pcsk5	1.322356161	Tmem116	-1.979690154
Sypl2	1.322081379	Slc45a3	-1.975895607
Prr18	1.321870833	Pde9a	-1.97520147

Nrtn	1.317885922	D7Ertd443e	-1.974024611
Klf10	1.316083338	Ptgr1	-1.972617345
Rgl3	1.315301764	Bcl2l15	-1.972022737
Ndrg2	1.307143767	Il4	-1.97046556
Upp1	1.304609845	Reln	-1.967235443
Kcnh1	1.302562113	Tspan17	-1.962986558
Mgst3	1.301573935	Sh2d4b	-1.962668299
Wtip	1.300883077	Fam163a	-1.955736252
1110065P20Rik	1.300857275	Emp1	-1.955120457
Arhgap25	1.29442486	Loxl1	-1.954656243
Inha	1.293920199	Nlrc5	-1.953094508
Ascl2	1.29296259	Stmn4	-1.948641099
Cebpb	1.292360641	Ncam2	-1.947796132
Fam151a	1.29222034	Scn9a	-1.947582452
Krt80	1.290045998	Adamts2	-1.944646844
Klrk1	1.290042832	Dnajb7	-1.941569626
Catsper3	1.290042784	Cnn1	-1.941039375
Nme8	1.290042549	Pyroxd2	-1.939055595
Gabrp	1.290042338	Ahr	-1.936249472
Igfn1	1.290040751	Pdlim2	-1.935952913
Gm5111	1.290040592	Stk32a	-1.934571794
Cdcp1	1.290040241	Adgb	-1.9304823
Tsga13	1.29004012	Mx2	-1.930111402
Dpp4	1.290038803	Folr2	-1.927818291
Fam83a	1.290038682	Btc	-1.927093451
1700125H20Rik	1.290038483	Galnt12	-1.926625422
Ikzf3	1.290036643	Arhgap11a	-1.926359266
Psg28	1.290035473	Irx1	-1.92569898
Mecom	1.28723958	Kif4	-1.923686246
Ptgis	1.286542016	Esyt3	-1.917583164
Srm	1.284647291	Capn5	-1.915448511
Kcnk1	1.28341516	S100a10	-1.914951068
Arl5c	1.281638996	Tbx3	-1.913840517
Tlr4	1.280531983	Chrna3	-1.913081601
Pnp	1.280151631	Gnrh1	-1.903843121
Abhd16b	1.279733762	Gapt	-1.902321771
Ngef	1.278721475	Tbx19	-1.902315057
Agap2	1.276689606	Ccr4	-1.902314163
Il10ra	1.276215174	Sucnr1	-1.898484387
Cpvl	1.274268762	Gap43	-1.892477786
Rpl39l	1.274266177	Zfp872	-1.891850944
1810011H11Rik	1.274263661	Plxna4	-1.8912937
Parvg	1.273756715	Cd82	-1.888215862
1700001O22Rik	1.272621143	Hrasls	-1.885058043
Lepr	1.270229058	Grpr	-1.883402841
Fbll1	1.265224996	Olfr1361	-1.883400127
Bcl2a1b	1.264527309	Pla2g4f	-1.883398239
Prg4	1.263321319	Hao1	-1.883396659
Tceal7	1.263217265	Hsd11b2	-1.883292963
Eno2	1.262718541	Adam5	-1.883246597
Ablim3	1.259661525	Il4ra	-1.88006219
Slpi	1.257799816	Mtfr2	-1.87894722
Nlrp6	1.254576638	Hist1h2bm	-1.877037075
Trpc7	1.252457788	Cdca2	-1.876672908
Ltc4s	1.250092827	Col5a1	-1.875522985
Cort	1.247102583	Kif6	-1.872157451
Cd37	1.246604362	Abca6	-1.87065008
Itih3	1.243381739	Stx11	-1.870583951

Zic3	1.243264841	Atoh8	-1.869845185
Hist1h2bp	1.242960616	Zfp488	-1.866954988
Rerg	1.24127818	Rgs9	-1.866226337
Nog	1.239963405	Iapp	-1.866212899
Tmem82	1.238710922	Efna1	-1.866006056
Wdr72	1.238259264	Pik3cd	-1.865535074
Ptk2b	1.237251566	Hand1	-1.864235624
Rnd1	1.232261754	Acox2	-1.864234457
Dkk1	1.231261356	Sis	-1.864232841
Caly	1.228446199	4933403O08Rik	-1.864232772
Cox6a2	1.225429052	Gm15217	-1.864231793
Mboat4	1.225428965	Il13	-1.864230486
Apoa1	1.225424277	Cxcl9	-1.864229332
Comtd1	1.2248908	Gp5	-1.863733541
Raver2	1.222667771	Tac4	-1.863433966
Ppp1r14b	1.2222665	Smim5	-1.863427946
Gpd1	1.219446606	Rnaset2b	-1.862932154
Erf	1.218243088	Cpm	-1.862826586
Kcns1	1.218130837	Mak	-1.857544049
Mctp1	1.21668618	Dlgap5	-1.853931847
Cnih3	1.215178455	Csgalnact1	-1.853044895
Rom1	1.213391189	Srpk3	-1.851410178
Psd	1.213046979	Tal2	-1.850042283
1700029I15Rik	1.212887227	Dock9	-1.849513525
Aldoc	1.211812197	Btn1a1	-1.846081827
Rfx4	1.211678049	Fcgbp	-1.846081237
Lxn	1.211353611	Odf4	-1.846080738
Lor	1.209403093	Ggt6	-1.844802771
Trpc6	1.206580911	Kif23	-1.844483848
Arg2	1.206301281	Tenm1	-1.842693955
Rhbdl1	1.205749482	Fat4	-1.842143697
Hist1h2bk	1.203959414	Man1c1	-1.838273564
Klf16	1.19956902	Spn	-1.837510423
Cftr	1.198395876	Cst7	-1.837506653
Polr2f	1.197528596	Slc37a2	-1.83675372
Il1a	1.197370802	Nuf2	-1.835916123
Vnn3	1.197068153	Tlr5	-1.834994821
Gm826	1.197066324	Clcf1	-1.834112923
Meox1	1.197066225	Cep128	-1.833613281
Junb	1.195691403	Slc41a3	-1.83123793
Inpp5d	1.193689382	Ldlrap1	-1.829145672
Lalba	1.189972833	Tmem63a	-1.825222995
Cpa6	1.189971603	Mtnr1a	-1.825118678
Ptprcap	1.187061454	Bik	-1.825111749
Dusp6	1.18705629	Batf	-1.825109995
Pik3c2g	1.186316475	Ace	-1.822905737
Lrit1	1.18631432	Col6a1	-1.821603321
Gipc3	1.186313785	Echdc3	-1.820201558
C1qtnf4	1.184632038	Itga7	-1.819657484
Nr2f6	1.184067868	Abca5	-1.817488137
Pdia2	1.18258397	Sag	-1.817420867
Rtp1	1.182359234	Postn	-1.817385747
2610528A11Rik	1.181344002	Aldh1l2	-1.816840149
Cyp2s1	1.178059555	Hgfac	-1.816392317
Ascl4	1.177727786	Layn	-1.815265604
2310039H08Rik	1.177504254	Serpinf1	-1.81469698
Cygb	1.17656956	Krt73	-1.812481214
Upb1	1.174889495	Slitrk6	-1.810510935

Lmx1b	1.174887675	Anxa1	-1.807525471
C8a	1.17488599	Pvr	-1.807483486
Tgtp1	1.174885966	Lct	-1.807159476
Cyp1a2	1.174885058	Capn6	-1.8069022
Fgf16	1.174884174	Plekha6	-1.805644511
C1ql4	1.17488375	Dst	-1.802011339
Gm8909	1.174883292	Ermap	-1.801153341
Mroh5	1.174693487	Arhgef39	-1.800596528
Glul	1.172337497	Ces5a	-1.797220944
Prr22	1.171605824	Plscr4	-1.796363955
Kcng2	1.168564325	Itih5	-1.795246633
Fpr1	1.167017599	Pex5l	-1.793374592
Akap3	1.167008917	Lrriq1	-1.789964443
Golph3	1.165148358	Prr19	-1.789059502
Cnih2	1.161764942	Nid1	-1.787969321
Asic4	1.161693006	Sdsl	-1.787757565
Vstm2a	1.160660171	4933408B17Rik	-1.786487895
Tlr2	1.160214331	Arhgap6	-1.785650395
Pde1b	1.159946644	Upp2	-1.784019941
Aanat	1.157234194	Tubb4a	-1.782424397
Phgdh	1.156475589	Mroh2b	-1.781529356
Fam174b	1.15451938	Cryl1	-1.779515767
Lyplal1	1.154291735	Gdf6	-1.77812104
Chchd7	1.154055411	Qrfp	-1.776900493
B3gnt3	1.153924094	Snx30	-1.776573115
Foxe1	1.148638328	Sec14l5	-1.776075968
Prss56	1.148229216	Cldn20	-1.775793399
1110032F04Rik	1.14671658	Adra2b	-1.7744651
C1qb	1.1464117	Dock5	-1.773304384
Tmem238	1.145615901	Gabra3	-1.772561127
Kcnj16	1.144888243	Ifih1	-1.770285248
Snta1	1.14462141	Ankle1	-1.768571581
Arhgdib	1.144260859	P2ry2	-1.767864626
Gm14295	1.143308377	Cybrd1	-1.767548008
Scg2	1.142773793	Syngr4	-1.767261549
Espn	1.142387346	Hist1h4b	-1.764446483
C2	1.14171869	Ltbp1	-1.763028814
Kank3	1.140952789	Ppp1r3b	-1.76296588
Pus7l	1.138384879	Kcna6	-1.762440605
Cacna1e	1.137789274	Efemp1	-1.760317129
Crybb1	1.136492882	Rbms1	-1.75978034
Rbp7	1.13576504	Car6	-1.759140524
Cilp2	1.133552759	Cntn2	-1.758524688
Rapgefl1	1.132168038	Ptprt	-1.758336272
Klhdc8b	1.129723024	Lrp2	-1.757811671
Mical1	1.127230822	Smad6	-1.755324664
Il7	1.125863934	Mss51	-1.754104764
Rrp9	1.123587495	Itga1	-1.753498877
Hist2h3c1	1.123147039	Mapk13	-1.753356538
Stpg2	1.122962987	Igfbp5	-1.753086434
Ppara	1.122265364	Spef2	-1.752359612
Stxbp6	1.12036109	Fank1	-1.749624014
Spock1	1.12010401	Glb1l2	-1.747291976
Ltb4r2	1.120065969	Lrguk	-1.745852958
Aass	1.119165482	Syt4	-1.745168812
Il1rn	1.118646843	Tiam2	-1.742320863
Tcerg1l	1.117246857	Cacna2d2	-1.739673415
Mfsd2a	1.113868826	Pmch	-1.739193183

Traf4	1.113388796	Igsf10	-1.737658204
Zbtb7c	1.113074196	Trim43c	-1.735894795
Il15ra	1.110692538	Il11	-1.735791513
Fxyd2	1.110635475	Crispld1	-1.735190106
Trmt61a	1.109358405	Ddx60	-1.733192592
Mrpl12	1.107451576	Itgae	-1.731044154
Gm5617	1.106720915	Styk1	-1.728897941
Pip5k1b	1.105605339	Otoa	-1.727228636
Rit2	1.105517435	Col6a2	-1.727030343
Trem2	1.104083784	Adam32	-1.726743238
Pdzd2	1.102754941	Col6a3	-1.725770288
Zfp456	1.1018819	Cd44	-1.725349089
Wipf3	1.101663617	Fzd6	-1.719980591
Gjd2	1.100968574	4930562C15Rik	-1.719383863
Pp2d1	1.10087623	Sh3pxd2a	-1.716607738
Gm21949	1.10009815	Scd3	-1.716048286
Lcp2	1.099456551	1190007I07Rik	-1.715152462
Adam33	1.097686941	Colec11	-1.712780285
Tmprss11g	1.097686	Pcsk9	-1.712472979
Gdf3	1.097684561	B430306N03Rik	-1.709960739
Prdm12	1.097683583	Tcf23	-1.709959824
Zbbx	1.097681161	Rad51ap2	-1.709959259
Card10	1.097071677	Olfr1318	-1.709958264
Tusc1	1.096588048	Ffar3	-1.709956693
Olfml1	1.096379732	Olfr95	-1.709953742
Cntnap5a	1.096109368	Tead4	-1.708155591
Adprhl2	1.095868956	Kank2	-1.704291984
Pdzd9	1.094282175	Mlf1	-1.703905143
Zcchc12	1.091374858	Map1b	-1.700692368
Trim65	1.09117345	Bmp8b	-1.700535773
Dusp2	1.090779525	Chodl	-1.697490262
Ccl2	1.089986916	Ubc	-1.692132394
Gm2a	1.089632695	Rorc	-1.691927578
Scube1	1.08830953	Ctsk	-1.691881859
Hapln4	1.087321526	Vwc2	-1.691462377
Rln1	1.086118365	Enpep	-1.691045802
Npy1r	1.085558519	Ecscr	-1.691043982
Tcp10c	1.08308805	Pax3	-1.691041405
Cited1	1.079978607	Cyp4b1	-1.691040279
Tyrobp	1.079510275	Abcc9	-1.691037311
Jakmip1	1.078998346	Tdh	-1.688031576
Pdk4	1.078991364	Tmem217	-1.688027539
Laptm5	1.077348344	Prph	-1.684742684
Zdbf2	1.076983066	3632451O06Rik	-1.684378789
Olfm3	1.076715523	Plin1	-1.681491532
Tdrd9	1.075586803	Cit	-1.681386519
Prdx6	1.075289387	Vmn1r32	-1.680749331
Dhh	1.075229998	S1pr3	-1.679624871
Tmem121	1.074643027	Morn3	-1.679440964
E030030I06Rik	1.074480054	Vwa2	-1.677090916
Tmem240	1.074105852	Tnr	-1.675118831
Ubiad1	1.073610773	Mmp2	-1.67390703
Grin1	1.072358541	Macc1	-1.673799205
Ccdc69	1.071738222	Grm7	-1.673229071
Itgb2	1.06951795	Rhbdl2	-1.67290435
Fli1	1.068989543	Irs4	-1.672753656
Endog	1.068893916	Atp13a5	-1.67188119
Schip1	1.067163912	Cdx2	-1.671877604

Slc38a3	1.066992652	Serpinb1c	-1.671876502
Naf1	1.062683542	Mrap	-1.671875581
Vstm4	1.062306562	Gm364	-1.671873195
Timm8a1	1.062208494	Abca17	-1.671873103
Syt1	1.061688023	Lbp	-1.668751359
Dnajc22	1.059891052	Tmtc2	-1.66733181
Tomm20l	1.058452875	Myo1e	-1.667106773
Sdc4	1.058184985	Pml	-1.666256496
Foxr2	1.057766256	Csf3	-1.666064638
Cited4	1.056382318	Tulp1	-1.666064441
Ppil6	1.056262246	Abca12	-1.666063758
Tlr9	1.056160294	Cd55	-1.665827399
Car2	1.052193254	Cmbl	-1.664655709
Cd101	1.052183839	Idi2	-1.664002011
Fth1	1.050185308	AF529169	-1.662788765
Sh2d7	1.049725432	Tmem235	-1.662196929
Cox18	1.049510739	Map3k7cl	-1.661130501
Lrrc73	1.048915743	Aox4	-1.659776706
Crhbp	1.04814641	Sstr4	-1.659589528
Sema4a	1.046883324	Angptl7	-1.659555886
Etl4	1.046756317	Sox10	-1.659043725
Ube2l6	1.045333596	Aim2	-1.658959564
Hs3st2	1.045300939	Hspg2	-1.658640546
Rasd2	1.044977365	Ttc12	-1.658613591
Cfh	1.043077929	Lama4	-1.655919482
Crot	1.042344501	Isg20	-1.654181459
Foxp1	1.041665479	Olfml2a	-1.652504729
Bcar3	1.041452162	Il21	-1.652453647
Fam227b	1.040455756	Spata19	-1.652453431
Ccdc155	1.040363085	Cnga2	-1.652448552
St3gal6	1.038215878	1810062G17Rik	-1.652448051
Lgals9	1.03623318	Spata20	-1.652447605
Sft2d1	1.03616714	4833420G17Rik	-1.651197086
Gipr	1.033542047	Nrap	-1.650492666
Dmrta2	1.033491289	Spats1	-1.649588715
Ppp2r5a	1.032399571	Ciita	-1.645437535
Lcp1	1.032163055	Klf1	-1.644987545
Ptpn6	1.03172251	Epyc	-1.643756085
Cx3cr1	1.031678771	Hist1h2ab	-1.643042588
Mypn	1.03030879	Itga5	-1.641403048
Tulp2	1.028822477	Lrrc8b	-1.637939927
Rspo3	1.028720854	Nhs	-1.635692932
St6galnac6	1.028578582	Wisp3	-1.635178288
Slc25a48	1.027806526	Hist1h4m	-1.634596546
Hhipl2	1.027097608	Kif2c	-1.633734193
Atp2a3	1.026051984	AU022751	-1.632036949
Lemd1	1.024741085	Sema3e	-1.631741924
Gm14403	1.024504232	Shisa6	-1.630143171
Htra2	1.02371048	Kntc1	-1.62929213
Ehf	1.021601342	Ptpn22	-1.627494937
Lrrc10b	1.021460622	Vgll2	-1.627075798
Bdnf	1.018742053	Best2	-1.626543332
Kctd16	1.018203033	Nusap1	-1.622724128
Panx2	1.017843446	Fmn1	-1.622501782
Pld2	1.016597147	Tmem108	-1.620815019
Frmd7	1.015926341	Arid5b	-1.620564059
Spry4	1.014436543	Cyp2r1	-1.61735635
Sptb	1.012915249	Prrg3	-1.617037669

Gm9958	1.012573699	Flrt1	-1.614976233
Trib1	1.010630274	Emp2	-1.614206255
4933425L06Rik	1.00955282	Matn2	-1.6130492
Osgin1	1.008467913	Ppp1r17	-1.612846884
Cyp4f18	1.007104041	Slc22a21	-1.611549905
Ndst3	1.006125233	Spata21	-1.611161578
Kcnt1	1.005847806	Galm	-1.610950944
Ndufaf4	1.005274984	Strc	-1.610288051
Hcst	1.004117867	Hoxa3	-1.608897537
Cntnap3	1.004114891	Plce1	-1.606783035
Cdh26	1.002839476	Arhgef15	-1.60466283
Slc6a9	1.001698714	Mc5r	-1.604257827
Gpr82	1.001662637	Fam71d	-1.604144936
Hrh3	1.00104476	Pcdh9	-1.603650461
		Phldb3	-1.602738157
		Cyp1b1	-1.601491091
		Spon2	-1.600802393
		Gpr182	-1.600404981
		Syt13	-1.600175391
		Pctp	-1.599749725
		Hmgb2	-1.598784039
		6330409D20Rik	-1.597963337
		Fmod	-1.5973336
		Stk33	-1.596029421
		Slc22a3	-1.59590918
		Pi4k2b	-1.59529508
		Incenp	-1.591984072
		Ust	-1.590815038
		Pdzrn3	-1.589170625
		Srpx	-1.58864902
		St6gal2	-1.588371011
		Ereg	-1.587948168
		Entpd8	-1.585679235
		BC028528	-1.585674849
		Bglap	-1.585306361
		Slc24a1	-1.585164437
		Unc5c	-1.584479561
		Gen1	-1.582994344
		Mfsd7a	-1.582566542
		Vmn2r1	-1.581987826
		Ect2	-1.581589734
		Cenpl	-1.576205923
		Cd109	-1.575159605
		Tppp3	-1.574591057
		Bub1	-1.574586614
		Pabpc1l	-1.574197411
		Cdh6	-1.573965175
		Tmem255a	-1.572451869
		Dnajb13	-1.572206814
		Adamts1	-1.57156028
		Prr15	-1.571156382
		Jakmip3	-1.570679262
		Map3k8	-1.56771999
		Cartpt	-1.566680875
		Scara5	-1.566222961
		Cabp5	-1.563442259
		Bub1b	-1.562630478
		Vmn1r90	-1.56231073

		Bmp4	-1.56088832
		Zfp750	-1.560311552
		Htr3a	-1.558001327
		Zfp811	-1.557451534
		Pkd2	-1.557247821
		Hmgb4	-1.557158351
		Serpinb8	-1.556913716
		Gucy1b2	-1.556905416
		Pcdh20	-1.555909617
		Hrct1	-1.555568714
		Hrc	-1.553356496
		En1	-1.553340247
		Adcy10	-1.548391992
		Scube2	-1.547119607
		Pram1	-1.545422946
		Zfand4	-1.54389002
		Chrnb4	-1.541886511
		Stab2	-1.541417694
		Arhgap19	-1.540406894
		Vgll3	-1.540107852
		Dcc	-1.539867674
		Tmem45a	-1.539139533
		Crct1	-1.538327987
		Rnf152	-1.537263392
		C4b	-1.537069034
		Pcdh17	-1.536717794
		Aldob	-1.535953626
		Sgcd	-1.535223419
		Ube2c	-1.532230228
		Erich2	-1.531178871
		Elf4	-1.530192099
		Col4a1	-1.528289451
		Mme	-1.526515603
		Tmem53	-1.525350044
		Hist1h2ag	-1.52514875
		Mmp28	-1.523910168
		Acot12	-1.523631046
		Col1a2	-1.523375204
		Tmem239	-1.523034163
		Col6a4	-1.523007372
		Ccnf	-1.522679776
		Kif20b	-1.522279988
		Nkx6-2	-1.520870658
		Slco2a1	-1.519445741
		Ppp1r32	-1.518723797
		Col4a5	-1.517677739
		Espl1	-1.514229009
		Adamtsl3	-1.513307936
		Tmeff2	-1.512817147
		Aurka	-1.510577319
		Cd160	-1.50720988
		Anln	-1.506531038
		Pcsk6	-1.505024616
		Rnase4	-1.504409343
		Gpr17	-1.503603634
		Miip	-1.503313019
		Top2a	-1.501805458
		Rin1	-1.500752114

		Sycp2	-1.499866997
		Lrrc8c	-1.499530501
		Uba1y	-1.499032879
		Gm1141	-1.49898099
		Fitm1	-1.495673492
		Fancd2	-1.49494176
		Sytl1	-1.493950887
		Fam198b	-1.493905572
		Edn1	-1.493123588
		Fbxo5	-1.487879443
		Susd1	-1.487610115
		Kcnt2	-1.487346345
		Adam34	-1.487286785
		Dupd1	-1.487279766
		Slc46a2	-1.487279444
		Ern2	-1.487279256
		Hus1b	-1.487277932
		Cuzd1	-1.487277523
		Slc5a4b	-1.487277226
		Apon	-1.487176349
		Pde11a	-1.487158271
		Sema4d	-1.486928624
		Tmprss6	-1.486178282
		AI182371	-1.486143756
		Ndc80	-1.485653108
		Emp3	-1.481574103
		Kdr	-1.481520547
		Ccdc36	-1.481458056
		Epha2	-1.478024584
		Cd27	-1.477849647
		D6Ertd527e	-1.477849212
		Tep1	-1.477176788
		Col3a1	-1.477095867
		Melk	-1.476873265
		Mb	-1.474690208
		Dio2	-1.47410394
		1700123K08Rik	-1.473708723
		Dmbx1	-1.473705699
		Depdc1b	-1.473030395
		Serpinf2	-1.470477722
		Bcam	-1.470183963
		Dnaic2	-1.469258044
		Armcx4	-1.469111913
		Ago3	-1.468968421
		Rtkn2	-1.468050535
		Pcdhb2	-1.467767303
		Zan	-1.467603199
		Parpbp	-1.467422086
		Hmmr	-1.466930534
		4930452B06Rik	-1.466913778
		Sdr42e1	-1.466305074
		Zdhhc23	-1.466284266
		Pdlim1	-1.466166695
		Fry	-1.466157816
		Plk1	-1.46476716
		Tpx2	-1.463786047
		Fam46b	-1.462147259
		Aspa	-1.462003965

		Rassf9	-1.462003547
		Lrrc17	-1.462002107
		Hsd3b6	-1.462001174
		Avpr1b	-1.462001098
		Ush1c	-1.462000689
		Vmn2r57	-1.46200065
		Tgm6	-1.462000637
		Wfdc12	-1.462000401
		Ropn1	-1.461999679
		Fam129a	-1.459861104
		Anxa11	-1.459553005
		D430041D05Rik	-1.459378315
		Ephb1	-1.455719418
		Lgi3	-1.455021901
		Edil3	-1.454355926
		Epha4	-1.454200479
		Krt24	-1.453475722
		Fgl2	-1.45298698
		Mmrn2	-1.452907728
		Htr2c	-1.452580521
		Calhm2	-1.451651368
		4932443I19Rik	-1.450370451
		Nkx2-1	-1.450311061
		Il17ra	-1.449958152
		AI467606	-1.449196217
		Pcdh12	-1.449195508
		Trim43b	-1.449194024
		Adam21	-1.449170649
		Sdk1	-1.44901769
		Hmgcll1	-1.449017044
		Edar	-1.448723643
		S100a6	-1.447653125
		Hs3st6	-1.446812223
		Adamts14	-1.446167045
		Thsd1	-1.445067568
		Ankmy1	-1.444361048
		Olfml2b	-1.443482741
		Kcnq3	-1.443241452
		Muc15	-1.443012104
		Kcnh7	-1.442771144
		Kifc1	-1.442654516
		Lmod1	-1.440834753
		Agbl4	-1.439384613
		Utrn	-1.439286641
		Slc12a2	-1.438553006
		Ncapd2	-1.438275569
		Tmsb15b2	-1.43813951
		Esrrg	-1.437862289
		Sfn	-1.436990942
		Gm5475	-1.436274731
		Wdr86	-1.436274304
		I830077J02Rik	-1.436273703
		Tldc2	-1.436272597
		Pla2g2e	-1.436271993
		Cdh15	-1.436270881
		Card11	-1.436269321
		Nox4	-1.434923188
		Lrrk2	-1.432776032

		Kcnk7	-1.431592877
		Adam19	-1.430305451
		Sec16b	-1.429612043
		Cep55	-1.428305663
		Asic3	-1.427114312
		Isoc2b	-1.427097537
		Golgb1	-1.42493677
		Esrp2	-1.424193932
		Qrich2	-1.424149199
		Ildr1	-1.423840887
		Pde8a	-1.423811501
		Ankrd28	-1.421835288
		Gnat2	-1.421237137
		Slc17a9	-1.420711043
		Tnfrsf23	-1.419870067
		Txnip	-1.419704102
		Prnd	-1.419346916
		Vasn	-1.418703111
		Myo7a	-1.416706733
		Nfe2	-1.416532827
		Nipal3	-1.416100857
		Cd209c	-1.415182879
		Ido2	-1.414315062
		Grk1	-1.4114333
		Dcdc2a	-1.411245081
		Ccdc171	-1.410915838
		Zp1	-1.410082276
		Pdyn	-1.410080174
		Nmu	-1.410078814
		Zscan10	-1.410078424
		Slc4a1	-1.41007838
		Psg29	-1.410077725
		Ly6a	-1.410077431
		Zfp704	-1.409783887
		Fbln1	-1.406776992
		Id1	-1.405755911
		Pon3	-1.402596101
		Raet1d	-1.402370415
		Ptgir	-1.401101287
		Greb1	-1.400573803
		Spag5	-1.399539045
		Mcmdc2	-1.398505129
		Lcor	-1.393455126
		Pak3	-1.393394542
		Zfp933	-1.3927209
		Nuak2	-1.391735429
		Atp6v0a4	-1.391351895
		Calr4	-1.391252783
		Psg23	-1.391086248
		Prr11	-1.390499497
		Sulf2	-1.3902749
		Nek2	-1.390062042
		Cobll1	-1.389571238
		Shroom1	-1.389539161
		Birc5	-1.389237349
		Nuggc	-1.387896776
		Cntn4	-1.387448127
		Gpr179	-1.387390833

		Ccdc110	-1.387081064
		4930486L24Rik	-1.386465074
		Zfp536	-1.386012509
		D630045J12Rik	-1.383017198
		Guca1b	-1.382927393
		Slc1a1	-1.382387352
		Pdcd1lg2	-1.381617658
		Gpr161	-1.380019612
		Tmsb15l	-1.378543283
		Tert	-1.377647357
		Zpld1	-1.377634128
		Crygs	-1.376849684
		Akap6	-1.375253314
		Tes	-1.375011778
		Heg1	-1.374263036
		Klf8	-1.374243075
		Slc16a8	-1.373093615
		Serinc2	-1.373001859
		Smoc2	-1.372689292
		Acta2	-1.372288629
		Slit1	-1.371993464
		Mfsd9	-1.371737286
		Olfr613	-1.370486851
		Lbx2	-1.369265849
		Herc6	-1.367940275
		Lhfpl2	-1.36791999
		Fat1	-1.365776705
		Rtn4rl2	-1.364267132
		Tmem178b	-1.363737566
		Ryr1	-1.36371717
		Cpa4	-1.36296864
		Hspb1	-1.362539612
		Tgfbr3	-1.362293235
		Slc25a24	-1.361752643
		Zfp941	-1.361578035
		Notch1	-1.361248267
		Knstrn	-1.359291044
		Agbl2	-1.358873841
		Npffr1	-1.357403439
		Eml2	-1.357378903
		Tmc1	-1.355393088
		Kif18b	-1.354749483
		Cbln2	-1.352398874
		Bmf	-1.352019587
		Syt14	-1.3512878
		Selp	-1.34981324
		Slc19a3	-1.349812837
		C1ql3	-1.34939858
		Best1	-1.347862967
		Astn2	-1.347064083
		Fzd5	-1.346871338
		Polq	-1.345465757
		Pde1a	-1.344827567
		Psmd9	-1.344820176
		Syne1	-1.344262974
		Macf1	-1.343614383
		Sema3d	-1.343467434
		Fam228a	-1.343060772

		Uba7	-1.34293325
		Zfp773	-1.342499964
		Prox1	-1.342359134
		Brdt	-1.340730548
		Acvrl1	-1.339487757
		Rps6ka1	-1.339235446
		Ephb2	-1.339064155
		4933402D24Rik	-1.338616408
		Pcdhb21	-1.338458919
		Hipk4	-1.334686466
		Slc27a5	-1.334151546
		Ebf1	-1.333779474
		Hydin	-1.33154862
		Thsd7a	-1.330654772
		Catsperd	-1.330071175
		Ttk	-1.329940467
		Nkx2-2	-1.32944973
		Cyp2j8	-1.328832
		Ccdc18	-1.328550985
		Socs3	-1.328507391
		Plin4	-1.328309819
		Lrg1	-1.328156222
		Slc44a5	-1.327724301
		Fv1	-1.327577598
		Cdh19	-1.327344031
		Rbm47	-1.327232057
		Inhbc	-1.327168752
		Cdk5rap2	-1.327029091
		H2-T24	-1.326412529
		Pcdhb15	-1.324718483
		Pdgfrb	-1.324135922
		Kif5a	-1.32392894
		Lrrc69	-1.323853972
		Nrcam	-1.323680831
		5730507C01Rik	-1.322994105
		Jag1	-1.322970909
		Ttll13	-1.322432799
		Esco2	-1.31925522
		Tet2	-1.318303092
		Akap9	-1.317023776
		Fam46c	-1.31677475
		Ston2	-1.314049278
		Trpc4	-1.313898252
		Il1rapl2	-1.31389066
		Ghrh	-1.313859673
		Cyfip2	-1.313567858
		Eml1	-1.313333574
		Slc38a6	-1.312848307
		G2e3	-1.311456449
		Mitf	-1.310747997
		Cdh11	-1.309003654
		Dixdc1	-1.307971189
		Itpr3	-1.306521756
		Plaur	-1.306507506
		A730017C20Rik	-1.306222597
		Pnma3	-1.305230862
		Ska3	-1.304455187
		Lctl	-1.303887955

		Brip1	-1.3037254
		Pot1b	-1.302860426
		Dok7	-1.302799263
		Nrp2	-1.301954408
		Prss8	-1.301527407
		Rpgrip1l	-1.301302634
		Strn	-1.300557271
		Lgals1	-1.298889344
		Syt2	-1.297064574
		Cep89	-1.29686319
		Cdca3	-1.29652914
		Poc1a	-1.295471955
		Gm6377	-1.294925776
		Defb20	-1.294922269
		Cxcl17	-1.294921783
		4931409K22Rik	-1.294920113
		Prps1l1	-1.294919626
		Rln3	-1.294917912
		Ppp3r2	-1.292348552
		Trim69	-1.288883287
		Hhatl	-1.288298975
		Galns	-1.287730846
		Loxl4	-1.287672625
		H2-Q4	-1.286060486
		Jsrp1	-1.286059204
		Cdkn3	-1.285647975
		Nox1	-1.28233825
		Fat2	-1.282338047
		Mid1	-1.28201619
		Erbb3	-1.281349867
		Cep112	-1.280773914
		Lrrn4cl	-1.280440068
		S100a2	-1.279269036
		Dbf4	-1.278016093
		Troap	-1.277864004
		Slc16a12	-1.277859857
		Islr2	-1.277756726
		Trpm3	-1.277665551
		Ccdc33	-1.276623183
		Qpct	-1.27633802
		Fndc9	-1.272972633
		Cd1d1	-1.272769327
		Cenpp	-1.269960365
		Mslnl	-1.269647612
		Atp4b	-1.269645983
		Tmem182	-1.269645043
		Fasl	-1.269644804
		Olfr1420	-1.269644736
		Ugt2b37	-1.269644278
		Dsc1	-1.269643988
		Olfr90	-1.269643152
		Mrgprx2	-1.269641946
		Kif22	-1.268295991
		Mis18bp1	-1.266655719
		Dnaaf3	-1.263563144
		Cdc25c	-1.262450712
		Gjb3	-1.261118922
		Pecam1	-1.260615041

		Ltb	-1.259844661
		Sned1	-1.259479844
		Slc10a1	-1.258526143
		Ptprj	-1.257353805
		Ano7	-1.256837361
		Papln	-1.256240993
		Kazn	-1.255426792
		Kcnq1	-1.254146427
		Adam4	-1.254020181
		Pth1r	-1.253404972
		Chrm2	-1.2532135
		Vdr	-1.253082643
		Ncapg	-1.252975844
		Crb2	-1.252862052
		Lrig3	-1.25110054
		Suco	-1.248085298
		Acss3	-1.247641309
		Cast	-1.24659427
		Zfp369	-1.245595862
		Mast4	-1.24556239
		Rsph4a	-1.245145711
		Slc23a1	-1.245108028
		Ccdc82	-1.244637962
		Anxa2	-1.244203972
		Vill	-1.243923651
		Arhgap8	-1.243917318
		Crip3	-1.242976238
		Hdac1	-1.24270228
		Tspan18	-1.242426117
		Prc1	-1.242162569
		1700022I11Rik	-1.241242303
		Racgap1	-1.241052825
		Mmp11	-1.24094393
		Atp6v1c2	-1.240667962
		Zfp618	-1.240402285
		Gcnt4	-1.240296406
		Sdk2	-1.236330605
		Cpne1	-1.235818607
		Lamc2	-1.23572863
		Ccng2	-1.235555583
		Decr2	-1.235544146
		Sorbs2	-1.234992411
		Syn3	-1.233518748
		Llgl2	-1.233264185
		Pcdh19	-1.232871573
		Rasgrp3	-1.232450216
		Slc26a5	-1.231430852
		Cxcr6	-1.231426781
		Sh3d19	-1.231305954
		Iqgap1	-1.231255756
		Best3	-1.230880502
		Prrg4	-1.230588586
		Hsf2bp	-1.227789894
		Abcb1a	-1.227789453
		Ckap5	-1.226926412
		Kcnj13	-1.226307687
		Stmn2	-1.225962765
		Gsta3	-1.224821909

		Hoxb5	-1.224246614
		Spdl1	-1.224074387
		Cchcr1	-1.223802177
		Col11a1	-1.222999091
		Opn3	-1.222870845
		Sec31b	-1.221877027
		Mylpf	-1.221661975
		Snapc1	-1.221506712
		Rad51b	-1.221433216
		Baz2b	-1.221053821
		Itgbl1	-1.220134529
		Flnb	-1.219868018
		Fam229a	-1.217723846
		Snx31	-1.217722457
		Serpini2	-1.217722224
		Sult5a1	-1.21772194
		Hsd17b14	-1.217720937
		Kctd14	-1.216205144
		Mnd1	-1.215818865
		Nxph3	-1.215267952
		Tcf7l2	-1.214737264
		Emb	-1.214429958
		Grhl2	-1.214187865
		Dusp23	-1.213954344
		Kcnmb1	-1.212258501
		Pcnt	-1.212032387
		Cdk19	-1.211214083
		Nek3	-1.210086956
		Unc13b	-1.208590087
		Aurkb	-1.207345303
		Kidins220	-1.207215012
		Mia3	-1.207155372
		Zkscan7	-1.206287714
		Lrrtm2	-1.20426901
		Odf2	-1.203433106
		Rictor	-1.203092313
		1700007K09Rik	-1.202580224
		Rgs16	-1.202576673
		Xkr7	-1.20252884
		Grik1	-1.202225388
		Evi2a	-1.20188828
		Slc12a7	-1.200488772
		Col11a2	-1.19983102
		Bnc2	-1.199341157
		Spata13	-1.197717881
		Manba	-1.194913729
		Tmod2	-1.19488446
		Wfikkn2	-1.194023061
		Bmp15	-1.193859019
		Epn3	-1.193856661
		Tbx21	-1.193856022
		Cntnap5c	-1.193854192
		Omp	-1.193810832
		Aph1c	-1.193343708
		Rnf122	-1.193319577
		Disp2	-1.193202295
		Tmem63c	-1.193081688
		Epha6	-1.192731626

		Npm2	-1.192345953
		Alas2	-1.192202533
		Tfcp2l1	-1.19204589
		Tenm2	-1.192016706
		Scube3	-1.191463138
		Pik3r3	-1.191104517
		Nlgn1	-1.191027685
		Acyp1	-1.18964793
		Slc43a3	-1.187349213
		1700020D05Rik	-1.186941805
		Camk4	-1.185497043
		Rab9b	-1.184108991
		Robo2	-1.183895002
		Rnf125	-1.183840187
		E2f7	-1.183704364
		Icosl	-1.183630601
		Pcdhb3	-1.182626493
		Creb3l4	-1.182587338
		Hcn1	-1.181516013
		Hist1h4c	-1.181010615
		Frmd5	-1.180731087
		Slc6a3	-1.179888359
		Plin5	-1.17910552
		Ecm1	-1.178823005
		Plag1	-1.178423514
		Dusp16	-1.178298095
		Ccna2	-1.178174141
		Zeb2	-1.177789452
		Flrt2	-1.177194514
		Cmah	-1.176885402
		Hcrtr2	-1.176881659
		Cdh18	-1.176811018
		Helb	-1.175078191
		Tmtc4	-1.173186711
		Henmt1	-1.172636222
		Fbln7	-1.169095286
		Ikbke	-1.168999678
		Runx1	-1.168422246
		Ckap2l	-1.168245784
		Agtrap	-1.168161998
		Bhlha15	-1.167383216
		Cobl	-1.167243539
		Piezo1	-1.167241841
		Zranb3	-1.167142072
		Gabrg3	-1.166458032
		Xrra1	-1.166272355
		Tnfaip2	-1.166069754
		Slc38a4	-1.165664266
		Cep350	-1.165041879
		Otud7b	-1.164590729
		Vat1l	-1.163972233
		Stil	-1.162903063
		Sorcs1	-1.162867337
		Oas3	-1.162822984
		Pla2g2f	-1.162819464
		Mfsd6l	-1.162818506
		Zfp819	-1.162818213
		A530016L24Rik	-1.16253565

		Hspb11	-1.161174404
		Rffl	-1.161085037
		Efcab11	-1.160927049
		Grin3a	-1.160205877
		Lgals3	-1.158955181
		Trim30d	-1.15813147
		Armc2	-1.157860247
		Gm14434	-1.157399984
		Gm4724	-1.157399984
		Pcdhb16	-1.156910851
		Shroom3	-1.156574659
		Gal3st1	-1.156545927
		Efnb2	-1.156391978
		D130040H23Rik	-1.15632073
		Tnnt1	-1.155661794
		Tmc3	-1.154751387
		Prox2	-1.15452822
		Anxa3	-1.154203184
		Fbn1	-1.154146531
		Cep72	-1.153456903
		Plxna2	-1.153432714
		Pdgfra	-1.152686803
		Kcns3	-1.151779444
		Pcdh7	-1.151746128
		Amhr2	-1.151439183
		Chrnb1	-1.150709324
		Fam161a	-1.150689456
		Poln	-1.149224464
		Atp2a1	-1.149124475
		Snai1	-1.148968517
		Sfi1	-1.14719006
		Cdkl2	-1.146873011
		Tgfb1i1	-1.146086376
		Cdca8	-1.146081334
		Cdr1	-1.1459257
		Zfp433	-1.145646511
		Fga	-1.14536821
		Myo1a	-1.144773693
		Cnr1	-1.144341227
		Catsperg1	-1.143309707
		Ttbk2	-1.143198918
		Npy	-1.143105872
		Gm6525	-1.141938203
		Slc16a7	-1.141548759
		Shcbp1	-1.140666721
		Ephb6	-1.140481551
		Rnf144b	-1.139883584
		Sez6l	-1.139808687
		Ccbe1	-1.139795665
		Cabyr	-1.139167594
		Mysm1	-1.137977389
		9130008F23Rik	-1.137816108
		Myh11	-1.137754251
		Xrn1	-1.136968707
		Sln	-1.136810549
		Hist1h3c	-1.136348303
		Sema4c	-1.13627214
		Cpxm2	-1.135740078

		Ccdc8	-1.135240824
		Ccin	-1.134811354
		Rttn	-1.133468934
		Htr1b	-1.133431106
		Eif4ebp3	-1.13321853
		Trp63	-1.1319584
		1700034I23Rik	-1.131749464
		Sv2b	-1.131624507
		Slc29a3	-1.131467744
		Rhbdf2	-1.131260174
		Zfp772	-1.131213943
		Cercam	-1.129841215
		Pnma2	-1.128677086
		Plp2	-1.128662374
		Il6ra	-1.128462597
		Stap2	-1.128097857
		Pou5f2	-1.128014882
		Artn	-1.127448846
		Fam83d	-1.127167721
		Capn1	-1.126852919
		Pak1	-1.126844306
		Nckap5	-1.126523837
		Rab11fip1	-1.126397652
		Micall2	-1.126006937
		Cldn1	-1.124339005
		Proca1	-1.124220163
		Trim30a	-1.123345601
		Ankrd34b	-1.123315721
		Ptprb	-1.12134348
		Gria4	-1.120871355
		Plekhg3	-1.12070862
		Zbtb37	-1.120230937
		Slc8a2	-1.119733089
		Sesn3	-1.119388337
		Ly6g6f	-1.118988581
		Spc25	-1.118945713
		Cep192	-1.117273337
		Ccdc148	-1.117216956
		Tnnc2	-1.116920038
		Bach2	-1.116394699
		Ada	-1.11624422
		Col1a1	-1.115763552
		Atp10a	-1.115548046
		Gdf5	-1.11534853
		Fam134b	-1.115314
		Dpysl4	-1.114846865
		Prdm10	-1.114553328
		Fam46a	-1.114028144
		Igf2r	-1.113982416
		Myom1	-1.113910954
		Olfr1349	-1.1136593
		Nid2	-1.113396333
		Ero1lb	-1.1129455
		Ninl	-1.111843229
		Dok6	-1.110927539
		Gpha2	-1.109747854
		Col4a3	-1.107429085
		Gng13	-1.107247716

		Usp40	-1.107174351
		Iqcc	-1.106825389
		Dnm3	-1.106700844
		4932438A13Rik	-1.106454646
		Slc37a1	-1.105951153
		Tapbpl	-1.104387149
		Rapgef5	-1.103958154
		Cep250	-1.103785086
		Psd2	-1.103581131
		Capsl	-1.103348318
		Cyp26c1	-1.103070231
		Cadm1	-1.102808809
		Ifnz	-1.102131574
		Clcn5	-1.101537253
		Zfp804b	-1.101529821
		Actn1	-1.100832289
		Galnt3	-1.100761923
		Gxylt1	-1.100715399
		Stx2	-1.100594708
		Cacnb2	-1.10025261
		Gm5415	-1.100238291
		Prss35	-1.100171078
		Zfp791	-1.099955181
		1700093K21Rik	-1.099586138
		Ang	-1.099367886
		Apobec2	-1.099258911
		Anpep	-1.098124354
		Myo6	-1.097827215
		Nradd	-1.097713146
		Neurl1b	-1.097212087
		Trip11	-1.097181817
		Ap1s3	-1.096781075
		Adamts18	-1.096529872
		Kcnj9	-1.09638918
		Tmem2	-1.095287958
		Naip1	-1.094478686
		Lrrc72	-1.094476762
		Spaca5	-1.094475465
		H2-DMb1	-1.094474925
		Glra4	-1.094473637
		Cpeb4	-1.092711103
		Scml2	-1.091833755
		Cep85l	-1.09112182
		Dach2	-1.09080411
		Ankrd53	-1.090509262
		St8sia3	-1.090188759
		Lrig1	-1.090074137
		Adamtsl1	-1.089657443
		Rprm	-1.089463165
		Smtn	-1.089340425
		Ctrl	-1.089247853
		Tekt2	-1.087419196
		Gcnt7	-1.087383071
		Ska1	-1.087229154
		4930550C14Rik	-1.086912963
		Hpse	-1.086837092
		Gal	-1.086779781
		Cd247	-1.086777494

		Rab33a	-1.08674746
		Tmem245	-1.086387866
		Slc8a1	-1.086126166
		Fam129b	-1.086095195
		Trim15	-1.085679117
		Ubxn10	-1.085512952
		Parm1	-1.084889085
		Chst3	-1.084694918
		Bora	-1.083561643
		Fam221b	-1.083142993
		Gsta1	-1.081102942
		Mcidas	-1.081100415
		Hipk2	-1.07948944
		Wfdc3	-1.079041653
		Tulp4	-1.078228912
		Cep152	-1.078091302
		Crb3	-1.077930165
		Pnp2	-1.077235214
		Ncapg2	-1.076981891
		Tab3	-1.075554454
		Efr3b	-1.075408289
		Gas2l3	-1.075234051
		Stard6	-1.074992598
		Abcb4	-1.074907879
		Atp8a1	-1.07410527
		Gng8	-1.073917145
		Tcf7l1	-1.073671333
		Pkn3	-1.073611451
		Foxf2	-1.073207916
		Psen2	-1.072794091
		Tspan15	-1.072160127
		Plch1	-1.071356968
		Pcdhb7	-1.071313116
		Reep4	-1.070892352
		Ggt5	-1.069989063
		Olfr267	-1.069987105
		Acr	-1.069956735
		Pls3	-1.069934699
		Gsg1	-1.068657938
		Phka2	-1.06730491
		Adamts19	-1.06707255
		Man1a	-1.065962872
		Nalcn	-1.063988225
		Nppb	-1.063435277
		Hebp2	-1.063231578
		Tstd3	-1.060972452
		Dusp4	-1.060184297
		Frem1	-1.060013685
		Gprin3	-1.059981128
		Pdcd4	-1.059337804
		Cnp	-1.059138293
		Podxl	-1.05911385
		Pdgfa	-1.058625578
		Lhx6	-1.058197212
		Trpv2	-1.057566402
		Lnpep	-1.056447173
		Map7	-1.055982284
		Csmd3	-1.055379263

		Hyls1	-1.055099386
		Pate4	-1.054897472
		Tecta	-1.054353557
		Grm1	-1.05263304
		Trim56	-1.051672386
		Npnt	-1.05076084
		Nsun6	-1.049890338
		Scarb2	-1.04967292
		Ccdc77	-1.049653997
		Dnajc6	-1.049565783
		Lcorl	-1.048507059
		Kif24	-1.048453131
		Mmp25	-1.047507185
		Fam160a1	-1.047323842
		Clec1a	-1.046964588
		Mab21l2	-1.046963675
		Mfsd6	-1.0469196
		Igfbpl1	-1.046552172
		Cd9	-1.046404052
		Mdm1	-1.045800085
		Unc5d	-1.045026353
		Secisbp2l	-1.044997186
		Atf6	-1.044147445
		Kif21a	-1.044139411
		Amz1	-1.043622847
		Obscn	-1.042843668
		Pros1	-1.042794717
		Gli3	-1.042337758
		Apold1	-1.042296888
		Mef2b	-1.041798534
		Dync1h1	-1.041533433
		Tacc3	-1.041370878
		5730480H06Rik	-1.041062461
		Celsr1	-1.041039074
		Pcdhb5	-1.040898383
		Pear1	-1.040881431
		Hist2h4	-1.039772473
		Rdh12	-1.039021311
		Dock6	-1.038723488
		4930503L19Rik	-1.038688233
		Ggta1	-1.037621111
		Cda	-1.036995896
		Tnfaip8	-1.035673858
		Gm7694	-1.033741126
		Nphp3	-1.032996109
		Lama3	-1.032069296
		Rdh16	-1.031888627
		Zbp1	-1.031886612
		4833427G06Rik	-1.031808544
		Kirrel3	-1.031076855
		Nnmt	-1.030884394
		Wnt5b	-1.029892173
		H2-M10.2	-1.029697368
		Fstl5	-1.029455654
		Hc	-1.029030434
		Fstl4	-1.028613214
		Gab3	-1.028242377
		Lif	-1.028023528

		Slc44a3	-1.027794319
		9530053A07Rik	-1.026697803
		Fbxo24	-1.026581592
		9030624G23Rik	-1.02631674
		Rdh5	-1.025875551
		Nudt12	-1.025825029
		1810041L15Rik	-1.025287612
		B3gnt7	-1.025119209
		Cps1	-1.02435007
		Cenpa	-1.023775422
		Sdcbp2	-1.023526429
		Herc1	-1.022652859
		Frem2	-1.022393786
		Ccdc50	-1.02235126
		Klhl28	-1.022289415
		Plcxd3	-1.022272588
		Eif2ak3	-1.02208401
		Wisp1	-1.022034526
		Slc27a3	-1.021849345
		Hdx	-1.021698158
		Als2cl	-1.021507875
		Sun2	-1.021313891
		Chml	-1.021003228
		Reck	-1.020812145
		Arrdc3	-1.02071469
		Pklr	-1.019614142
		D430019H16Rik	-1.019576623
		Pcdh15	-1.019521038
		Wnt2b	-1.01913201
		Ccnb2	-1.019084597
		Grik3	-1.01835419
		Klrg2	-1.017224418
		Spatc1l	-1.016774831
		Iqcj	-1.016772675
		1700019A02Rik	-1.016769496
		Atp7a	-1.01624918
		Eln	-1.016166486
		Lrig2	-1.016066585
		Cpd	-1.01557842
		Palld	-1.01524609
		Ccdc146	-1.01439719
		Slx4ip	-1.014322597
		Gabre	-1.014092863
		1700029H14Rik	-1.014091332
		Col5a2	-1.013795224
		Frrs1	-1.012543837
		Tgfbi	-1.012242495
		Gabrb2	-1.01217463
		Atl1	-1.012099934
		Scyl2	-1.01162434
		Mtus1	-1.011372059
		Tekt1	-1.011287623
		Duxbl1	-1.01117077
		Duxbl3	-1.011169759
		Duxbl2	-1.011169759
		Rtn4	-1.010422672
		3300002I08Rik	-1.010264393
		Zbed6	-1.009960212

		St8sia2	-1.009788351
		Anxa7	-1.00957039
		Tmbim1	-1.009244727
		Tgfbr1	-1.009142634
		Myh10	-1.009078683
		CK137956	-1.008866355
		Mansc1	-1.008843471
		Kif18a	-1.008667804
		Lmbrd2	-1.00844331
		Daam1	-1.008373426
		Xkr5	-1.008278653
		Acad12	-1.007910595
		Mroh2a	-1.007829601
		Cdc20	-1.007226401
		Grm5	-1.006914693
		Pcdhb19	-1.004841124
		Plau	-1.004265719
		Creb3l2	-1.004136056
		Ell2	-1.003829292
		Slc4a5	-1.003825839
		Tbce	-1.003770904
		Atp11b	-1.003149541
		Htr4	-1.002983016
		Cdc25b	-1.002612127
		Olfr70	-1.001499296
		Utp14b	-1.001002777
		Kif20a	-1.000756757

Supplementary Table 3. Primer sequences for qRT-PCR assay of target genes.

Gene	Forward primer	Reverse primer
Ccna2	TGGATGGCAGTTTTGAATCACC	CCCTAAGGTACGTGTGAATGTC
Ccnb1	GCCAAGAGCCATGTGACTATC	CAGAGCTGGTACTTTGGTGTTC
Ccnf	AGAGACTGAATACGGGTTCTGA	TCCCAAGCAGTGTAGTATGGAA
Ccng2	AGGGGTTCAGCTTTTCGGATT	AGTGTTATCATTCTCCGGGGTAG
Cdc25b	TCCGATCCTTACCAGTGAGG	GGGCAGAGCTGGAATGAGG
Cdc25c	GGCAAACCTAAGCATTCTGTCG	CCAGAGGTCCAGATGAATCCA
Cdca3	CTGAGCGAAGTATTGGAGACAG	CTGCGGATTGTTTGGCTTCC
Cdk19	GGTCAAGCCTGACAGCAAAGT	TTCCTGGAAGTAAGGGTCCTG
Cldn11	ATGGTAGCCACTTGCCTTCAG	AGTTCGTCCATTTTTCGGCAG
Enpp2	TTTGCACTATGCCAACAATCGG	GGAGGCACTTTAGTCCTGTACTT
Kif11	GGCTGGTATAATTCCACGCAC	CCGGGGATCATCAAACATCTG
Mbp	GCAGCCAGCACCACTCTTGA	CAGCCGAGGTCCCATTGTTC
Myrf	CCTGTGTCCGTGGTACTGTG	TCACACAGGCGGTAGAAGTG
Cnp	TTTACCCGCAAAAGCCACACA	CACCGTGTCCTCATCTTGAAG
Ugt8a	ACTCCATATTTCATGCTCCTGTG	AGGCCGATGCTAGTGTCTTGA
Plp1	CCAGAATGTATGGTGTTCTCCC	GGCCCATGAGTTTAAGGACG
Bcas1	AGAAGCGAAAGGCTCGGAAG	AGGGACAGAATAACTCAGAGTGT
Tet1	CATTCTCACAAGGACATTCACAACA	AGTAAAACGTAGTCGCCTCTTCCTG
β-actin	GGCTGTATTCCCCTCCATCG	CCAGTTGGTAACAATGCCATGT
Ccl22	AGGTCCCTATGGTGCCAATGT	CGGCAGGATTTTGAGGTCCA
Ccl2	TCAAACTGAAGCTCGCACTCT	GGGGCATTGATTGCATCTGG
Ccr2	ATCCACGGCATACTATCAACATC	CAAGGCTCACCATCATCGTAG
Cx3cr1	GAGTATGACGATTCTGCTGAGG	CAGACCGAACGTGAAGACGAG
Itpr2	CCTCGCCTACCACATCACC	TCACCACTCTCACTATGTCGT
Cacna1a	CACCGAGTTTGGGAATAACTTCA	ATTGTGCTCCGTGATTTGGAA
Cacna1c	ATTGTGCTCCGTGATTTGGAA	ACTGACGGTAGAGATGGTTGC
Cacna2d1	GTCACACTGGATTTTCTCGATGC	GGGTTTCTGAATATCTGGCCTGA

Cacnb4	TACCTGCATGGAGTTGAAGACT	TTCGCTCTCTCAAGCTGGATA
Cacng5	ACCTGGAAGAAGGCATAATCCT	CTATGGTAAAACAGCGTCCTCG
Atp2b1	AGATGGAGCTATTGAGAATCGCA	CCCTGTAACACGGATTTTTCCTT
Atp2c1	GCAGGCAGAAGAAGCACCAA	CCTAGTAACCAGCCAACCAAC
Slc8a1	CTTCCCTGTTTGTGCTCCTGT	AGAAGCCCTTTATGTGGCAGTA
