# Supplementary material for: Ten-eleven translocation 1 mediated-DNA hydroxymethylation is required for myelination and remyelination in the mouse brain
Source: Nat Commun. 2021 Aug 24;12:5091. doi: 10.1038/s41467-021-25353-5 (PMC8385008; doi:10.1038/s41467-021-25353-5)
Supplement: Supplementary file 3 — Description of Additional Supplementary Files [file 41467_2021_25353_MOESM3_ESM.docx]

Description of Additional Supplementary Files

Title: Supplementary Movie 1

Description: Intracellular Ca^2+^ oscillations in ATP-treated control OPCs in culture as reveal by Fluo4.

Title: Supplementary Movie 2

Description: Intracellular Ca^2+^ oscillations in ATP-treated *Tet1* cKO OPCs in culture as reveal by Fluo4.
