# Supplementary material for: Ten-eleven translocation 1 mediated-DNA hydroxymethylation is required for myelination and remyelination in the mouse brain
Source: Nat Commun. 2021 Aug 24;12:5091. doi: 10.1038/s41467-021-25353-5 (PMC8385008; doi:10.1038/s41467-021-25353-5)
Supplement: Supplementary file 6 — Reporting Summary [file 41467_2021_25353_MOESM6_ESM.pdf]

nature research

Corresponding author(s): Xianghui Zhao

Last updated by author(s): Jul 31, 2021

# Reporting Summary

Nature Research wishes to improve the reproducibility of the work that we publish. This form provides structure for consistency and transparency in reporting. For further information on Nature Research policies, see our Editorial Policies and the Editorial Policy Checklist.

## Statistics

For all statistical analyses, confirm that the following items are present in the figure legend, table legend, main text, or Methods section.

| n/a | Confirmed | |
|---|---|---|
| ☐ | ☒ | The exact sample size ($n$) for each experimental group/condition, given as a discrete number and unit of measurement |
| ☐ | ☒ | A statement on whether measurements were taken from distinct samples or whether the same sample was measured repeatedly |
| ☐ | ☒ | The statistical test(s) used AND whether they are one- or two-sided<br>*Only common tests should be described solely by name; describe more complex techniques in the Methods section.* |
| ☒ | ☐ | A description of all covariates tested |
| ☐ | ☒ | A description of any assumptions or corrections, such as tests of normality and adjustment for multiple comparisons |
| ☐ | ☒ | A full description of the statistical parameters including central tendency (e.g. means) or other basic estimates (e.g. regression coefficient) AND variation (e.g. standard deviation) or associated estimates of uncertainty (e.g. confidence intervals) |
| ☐ | ☒ | For null hypothesis testing, the test statistic (e.g. $F$, $t$, $r$) with confidence intervals, effect sizes, degrees of freedom and $P$ value noted<br>*Give P values as exact values whenever suitable.* |
| ☒ | ☐ | For Bayesian analysis, information on the choice of priors and Markov chain Monte Carlo settings |
| ☒ | ☐ | For hierarchical and complex designs, identification of the appropriate level for tests and full reporting of outcomes |
| ☒ | ☐ | Estimates of effect sizes (e.g. Cohen's $d$, Pearson's $r$), indicating how they were calculated |

*Our web collection on statistics for biologists contains articles on many of the points above.*

## Software and code

Policy information about availability of computer code

| | |
|---|---|
| Data collection | Confocal images and Calcium imaging were collected with FV10-ASW 04.02.01.20 software.<br>Electron micrographs were captured with JEM-1230 electron microscope (JEOL LTD, Tokyo, Japan) eqquiped with CCD camera and examined with its application software (832 SC1000, Gatan, Warrendale, PA).<br>The western blot band was captured with Tanon5200 imager.<br>Cell cytometry was detected by guava easyCyte6HT (Millipore).<br>The qRT-PCR was performed with Bio-Rad CFX96 Touch Real-Time PCR Detection System (Bio-Rad).<br>Clampex 10.6 was used for electrophysiological data acquisition.<br>The behavior data was recorded by Smart 3.0 software.<br>Sequencing was running on Illumina HiSeq 3000, HiSeq 2500 or NovaSeq PE150 sequencer. |
| Data analysis | All statistical analyses were performed in Prism 8.0 or SPSS 21.0.<br>The western blot images were quantified using ImageJ 1.52p software.<br>The flow cytometry was analyzed by ModFit LT 5.0 (Verity).<br>Relative mRNA expression was calculated by Bio-Rad CFX Manager.<br>Clampfit 10.6 was used for eletrophysiological data off-line analysis.<br>The behavior data was analyzed by Smart 3.0 software.<br>RNA-seq: reads were mapped to mm10 using TopHat 2.1.1 (http://ccb.jhu.edu/software/tophat/ index.shtml) and were then analyzed by DEGseq (1.39.0) package to identify differentially expressed genes. Gene ontology (GO) analysis was performed using ToppGene Suite (http://toppgene.cchmc.org) and Gene Set Enrichment Analysis (GSEA 4.0.1; http://www.broadinstitute. org/gsea/index.jsp).<br>hMeDIP-seq: Base calling were performed using Off-Line Base caller software (OLB V1.8). After passing Solexa CHASTITY quality filter, the clean reads were aligned to mm10 using BOWTIE software (V2.1.0). Aligned reads were used for peak calling by MACS v2 (http://liulab.dfci.harvard.edu/MACS. DhMRs between two groups were identified by diffReps (Cut-off: log2FC=1.0, p-value=10e-4). DhMRs were |

annotated by the nearest gene using the UCSC RefSeq and database of multiple databases integration.
Cut&Run-seq: Reads in FASTQ format were first subjected to quality control to assess the need for trimming of adapter sequences or bad quality segments. The programs used in these steps were FastQC v0.11.7, Trim Galore! v0.4.2 and cutadapt v1.9.1. The trimmed reads were aligned to the reference rat genome version Rn5 with the program BOWTIE v2.3.4.1. Aligned reads were stripped of duplicate reads with the program sambamba v0.6.8. Peaks were called using the program MACS v2.1.2 with the narrow and broad peaks mode for Cut&Run-Seq.

For manuscripts utilizing custom algorithms or software that are central to the research but not yet described in published literature, software must be made available to editors and reviewers. We strongly encourage code deposition in a community repository (e.g. GitHub). See the Nature Research guidelines for submitting code & software for further information.

## Data

Policy information about availability of data

All manuscripts must include a data availability statement. This statement should provide the following information, where applicable:

- Accession codes, unique identifiers, or web links for publicly available datasets
- A list of figures that have associated raw data
- A description of any restrictions on data availability

The transcriptome, DNA hydroxymethylation profiling and Cut & Run-Seq data generated in this study have been deposited in the Gene Expression Omnibus database under accession code GSE122838. Previously published and deposited data for hMe-Seal-seq of WT NSCs were extracted from GSE65994 and ChIP-seq of H3K27ac were extracted from GSE42454. The data supporting this study are available in the Article, Supplementary Information, or available from the corresponding authors upon reasonable requests. A reporting summary for this article is available as a Supplementary Information file. Source data are provided with this paper.

# Field-specific reporting

Please select the one below that is the best fit for your research. If you are not sure, read the appropriate sections before making your selection.

☒ Life sciences ☐ Behavioural & social sciences ☐ Ecological, evolutionary & environmental sciences

For a reference copy of the document with all sections, see nature.com/documents/nr-reporting-summary-flat.pdf

# Life sciences study design

All studies must disclose on these points even when the disclosure is negative.

| | |
|---|---|
| Sample size | Sample size was chosen in accordance with similar previously published experiments (PMID: 20223198,22037496 ) Sample sizes were determined based on previous similar studies and were indicated in the legend of each figure and supplementary Figure. |
| Data exclusions | No data were excluded from analyses. |
| Replication | All animal experiments were performed according to the n of each experiment. A (n=3) means that the experiments were collected in 3 mice in this group. For Electron micrographs, a (n=6) means 6 slides from 3 animals per group. qRT-PCR on cell cultures have been replicated three times in independent experiments. Experiments were replicated at least twice for all analyses to provide convincing results. All attempts at replication were successful. |
| Randomization | Animals were chosen randomly in the same litters based on correct genotypes. However, each experiment contained animals from at least two different litters to ensure that the differences between genotypes can be observed in mice from different litters. Experiments involving in vitro study were assured randomization through double-blind experiments. |
| Blinding | Experimenters were blind to animal genotypes for data collection and persons responsible for data analyze were blind to animal genotypes (Quantification of immunostainning, EM, qRT-PCR, Western blot, behavioral scoring and electrophysiology). |

# Reporting for specific materials, systems and methods

We require information from authors about some types of materials, experimental systems and methods used in many studies. Here, indicate whether each material, system or method listed is relevant to your study. If you are not sure if a list item applies to your research, read the appropriate section before selecting a response.

## Materials & experimental systems

| n/a | Involved in the study | |
|---|---|---|
| ☐ | ☒ | Antibodies |
| ☒ | ☐ | Eukaryotic cell lines |
| ☒ | ☐ | Palaeontology and archaeology |
| ☐ | ☒ | Animals and other organisms |
| ☒ | ☐ | Human research participants |
| ☒ | ☐ | Clinical data |
| ☒ | ☐ | Dual use research of concern |

## Methods

| n/a | Involved in the study | |
|---|---|---|
| ☐ | ☒ | ChIP-seq |
| ☐ | ☒ | Flow cytometry |
| ☒ | ☐ | MRI-based neuroimaging |

# Antibodies

| | |
|---|---|
| Antibodies used | Primary antibodies for immunostaining and westernblot:<br>Olig2 (Millipore, AB9610, 1:500), Sox10 (Santa Cruz, 17342, 1:50), TET1 (Genetex, 124207, 1:100), PDGFRα (Abcam, ab61219, 1:500), CC1 (Oncogene Research, OP80, 1:200), MBP (Abcam, ab7349,1:500 for IHC and 1:1500 for Western blot), 5hmC (Active motif, 39769, 1:1000), CNPase (Sigma, C5922,1:500), Ki67 (Abcam, ab16667), GFAP (Millipore, mAB360,1:1000), GST-pi (Abcam, ab53943,1:50), Nestin (Genetex, GTX630201, 1:500), DCX (Millipore, ab2253,1:500), Itpr2 (Millipore, AB3000,1:50), ALDH1L1 (Proteintech, 17390-1-AP,1:100 for IHC and 1:1000 for Western blot), NeuN (Millipore ABN78, 1:800 for IHC and 1:2000 for Western blot), PV (Abcam, ab11427, 1:200), SST (Millipore, AB5494,1:50), VIP(CST, 63269,1:200), Anti-BrdU (# G3G4, 1:40, DSHB, Iowa City, IA, 1:40), Olig2 (Millipore,MABN50,1:1000), DCX (Proteintech, 13925-1-AP,1:1000), H3S10P (Cell signaling technology, 9706,1:500), β-actin (Proteintech, 66009-1,1:5000), Tubulin (Abbkine, A01030,1:1000), GAPDH (Proteintech, 60004-1, 1:15000).<br>For hMeDIP-seq, 5hmC (Diagenode, C15200200).<br>For CUT&RUN-seq experiments, TET1 (Active motif, 61943).<br>For OPC immunopanning, hybridoma supernatant anti-Ran-2 (ATCC #TIB-119); hybridoma supernatant anti-GalC (PMID: 7045870); hybridoma supernatant anti-O4 (PMID: 6786942). |
| Validation | Following antibodies have been validated for immunostainning in publications:  Olig2 (Millipore, AB9610), PMID:25821912; Sox10 (Santa Cruz, 17342), PMID:26631469; TET1(Genetex, 124207), PMID: 28531272; PDGFRα (Abcam, ab61219), PMID:29507336, PMID:24615693; CC1 (Oncogene Research, OP80), PMID:24615693; MBP (Abcam, ab7349,1:1500), PMID:30848441, PMID:24615693; 5hmC (Active motif, 39769), PMID:24615693; CNPase (Sigma, C5922), PMID:24615693, PMID:24615693; Ki67 (Abcam, ab16667), PMID:30623969; GFAP (Millipore, mAB360), PMID:25808087; GST-pi (Abcam, ab53943), PMID:30236122; DCX (Millipore, ab2253), PMID:24880216; Itpr2 (Millipore, AB3000) PMID:22159127; ALDHL1 (Proteintech, 17390-1-AP), PMID: 31828740; NeuN (Millipore ABN78), PMID:31332372; Anti-BrdU (# G3G4, 1:40, DSHB, Iowa City, IA), PMID:30623969; H3S10P (Cell signaling technology, 9706), PMID: 24453992; Anti-PV(Abcam, ab11427) ,PMID:32541656; Anti-SST(Millipore, AB5494),PMID: 21309724; Anti-VIP(CST, 63269), PMID: 32667666.<br>Following antibodies have been validated for Western blot assay in publications:  Olig2 (Millipore MABN50), PMID:23872598; DCX (Proteintech, 13925-1-AP), PMID: 30662396; β-actin (Proteintech, 66009-1,1:5000), PMID:29133412; Tubulin (Abbkine, A01030), PMID:30698991; GAPDH (Proteintech, 60004-1, 1:15000), PMID: 30608882.<br>For hMeDIP-seq, 5hmC (Diagenode, C15200200) has been validated in publications: PMID: 26937014, PMID: 28437360.<br>For CUT&RUN-seq experiments, TET1 (Active motif, 61943) has been validated by the manufacturer "Application: ChIP-seq".<br>For OPC immunopanning, hybridoma supernatant anti-Ran-2 (ATCC #TIB-119), PMID: 24003195; hybridoma supernatant anti-GalC, PMID: 7045870; hybridoma supernatant anti-O4, PMID: 6786942. |

# Animals and other organisms

Policy information about studies involving animals; ARRIVE guidelines recommended for reporting animal research

| | |
|---|---|
| Laboratory animals | Conditional knockout mice in  C57BL6/J background, aging from embryonic stage (E13.5) to adult stage (P99) were used.<br>Tet1 flox/flox mice were from Shanghai Model Organisms (Cat# NM-CKO-00075, PMID: 23770080); Tet3 flox/flox mice were from Dr. Guoliang Xu in Shanghai Institute of Biochemistry and Cell Biology, CAS (PMID: 21892189); Olig1-Cre mice were for Dr. Richard Lu in Cincinnati Children's Hospital Medical Center (PMID: 15703389); NG2CreERT mice were from Jackson lab (stock number 008538, PMID: 21266410); Itpr2 flox/flox mice were from RIKEN BRC (RBRC 10293, PMID: 16195467).<br>Mice from either sex were used. All mice used in this study were kept under stable 12-hour circles of darkness and light in the respective facilities. Room temperature was kept between 20 - 24 °C and air humidity between 45 - 65% as documented in daily controls. |
| Wild animals | The study did not involve wild animals. |
| Field-collected samples | The study did not involve samples collected from the field. |
| Ethics oversight | Protocols approved by the Animal Care and Use Committee of the Fourth Military Medical University and were conducted in accordance with the guidelines for the care and use of laboratory animals. |

Note that full information on the approval of the study protocol must also be provided in the manuscript.

## ChIP-seq

### Data deposition

- [x] Confirm that both raw and final processed data have been deposited in a public database such as GEO.
- [x] Confirm that you have deposited or provided access to graph files (e.g. BED files) for the called peaks.

| | |
|---|---|
| Data access links<br>*May remain private before publication.* | Generated by this study:<br>https://www.ncbi.nlm.nih.gov/geo/query/acc.cgi?acc=GSE122838<br>Other datasets:<br>https://www.ncbi.nlm.nih.gov/geo/query/acc.cgi?acc=GSE65994<br>https://www.ncbi.nlm.nih.gov/geo/query/acc.cgi?acc=GSE42454 |
| Files in database submission | FOR RAW FILES:<br>RNA-Seq:<br>Con-RNA-1.read1.fq<br>Con-RNA-1.read2.fq<br>Con-RNA-2.read1.fq<br>Con-RNA-2.read2.fq<br>TET1CKO-OPC1.read1.fq<br>TET1CKO-OPC1.read2.fq<br>TET1CKO-OPC2.read1.fq<br>TET1CKO-OPC2.read2.fq<br>hMeDIP-seq:<br>OPC1_sequence.fastq<br>OPC2_sequence.fastq<br>CKO1_sequence.fastq<br>CKO2_sequence.fastq<br>Input_sequence.fastq<br>OL1_sequence.fastq<br>OL2_sequence.fastq<br>CUT & RUN-Seq:<br>OPC_IgG CUT & RUN_CKDL200168706-1a_HMGNGDSXY_L3_1.fq<br>OPC_IgG CUT & RUN_CKDL200168706-1a_HMGNGDSXY_L3_2.fq<br>OPC_IgG CUT & RUN_CKDL200168706-1a_HMGNGDSXY_L4_1.fq<br>OPC_IgG CUT & RUN_CKDL200168706-1a_HMGNGDSXY_L4_2.fq<br>OPC_TET1_CUT & RUN_CKDL200168707-1a_HMGNGDSXY_L3_1.fq<br>OPC_TET1_CUT & RUN_CKDL200168707-1a_HMGNGDSXY_L3_2.fq<br>OPC_TET1_CUT & RUN_CKDL200168707-1a_HMGNGDSXY_L4_2.fq<br>OPC_TET1_CUT & RUN_CKDL200168707-1a_HMGNGDSXY_L4_1.fq<br><br>FOR PROCESSED FILES<br>Tet1_RNA-seq_exp.txt<br>OPC1_Mus_musculus_MM10_hisat2_alignment.bed.gz<br>OPC2_Mus_musculus_MM10_hisat2_alignment.bed.gz<br>CKO1_Mus_musculus_MM10_hisat2_alignment.bed.gz<br>CKO2_Mus_musculus_MM10_hisat2_alignment.bed.gz<br>Input_Mus_musculus_MM10_hisat2_alignment.bed.gz<br>OL1_Mus_musculus_MM10_hisat2_alignment.bed.gz<br>OL2_Mus_musculus_MM10_hisat2_alignment.bed.gz<br>OPC_IgG_CUT & RUN.bed<br>OPC_TET1_CUT & RUN.bed |
| Genome browser session<br>(e.g. UCSC) | https://www.ncbi.nlm.nih.gov/geo/query/acc.cgi?acc=GSE122838 |

### Methodology

| | |
|---|---|
| Replicates | One replicate for TET1 CUT & RUN-seq in rat OPCs |
| Sequencing depth | OPC_TET1_CUT & RUN, pair end, read number: 160853129, read length: 150bp<br>OPC_IgG_CUT & RUN, pair end, read number: 167660681, read length: 150bp |
| Antibodies | TET1 (Active motif, 61943) |
| Peak calling parameters | Reads were uniquely mapped to genome using Bowtie2. Peaks were called using MACS (--shiftsize=75) and PeakSeq (fdr below 0.5%) |
| Data quality | Reads were high quality, unique reads were used for peak calling with FDR below 0.5% |

| | |
|---|---|
| Software | Cut&Run-seq reads in FASTQ format were subjected to quality control to assess the need for trimming of adapter sequences or bad quality segments. The programs used in these steps were FastQC v0.11.7, Trim Galore! v0.4.2 and cutadapt v1.9.1. The trimmed reads were aligned to the reference rat genome version Rn5 with the program BOWTIE v2.3.4.1. Aligned reads were stripped of duplicate reads with the program sambamba v0.6.8. Peaks were called using the program MACS v2.1.2 with the narrow and broad peaks mode for Cut&Run-Seq. |

# Flow Cytometry

## Plots

Confirm that:

- [x] The axis labels state the marker and fluorochrome used (e.g. CD4-FITC).
- [x] The axis scales are clearly visible. Include numbers along axes only for bottom left plot of group (a 'group' is an analysis of identical markers).
- [x] All plots are contour plots with outliers or pseudocolor plots.
- [x] A numerical value for number of cells or percentage (with statistics) is provided.

## Methodology

| | |
|---|---|
| Sample preparation | Immunopanning purified OPCs were collected by centrifugation at 1000 g and the cells were resuspended in 0.1 M PBS. Then cells were dropped into precooled 70% ethanol for fixation - 20 ℃ overnight. Following two wash steps in PBS and centrifugation for 5 min at 1200 rpm, cell were treated with RNase and then stained with PI using the DNA Content Quantitation Assay (Cell Cycle) (Solarbio) according to manufacturer’s instructions. |
| Instrument | Guava easyCyte6HT (Millipore) |
| Software | ModFit LT 5.0 software |
| Cell population abundance | After cleaning out the PI residual, the cells were added into 96 well plates at a density of 5 * 10 ^ 5 / ml, and a total of > 10 ^ 5 cells were detected. Red fluorescence area and width were used to determine the dispersed single cells. |
| Gating strategy | The gating strategy was set up using control group cells before sorting. The sorting strategy is as follows: on the first sort, the major cell groups is included by drawing a gate around cells with 1.0-7.5 K forward (FSC-HLin) and side (SSC-HLin) scatter. On the second sort, the single cell was gated in 3.0K-5.0K Red Fluorescence Width and 1.5K-6.0K Area. By looking at forward scatter vs width, we could exclude cellular debris and large cell masses. |

- [x] Tick this box to confirm that a figure exemplifying the gating strategy is provided in the Supplementary Information.
